# Synthesis and characterization of a green and recyclable arginine-based palladium/CoFe_2_O_4_ nanomagnetic catalyst for efficient cyanation of aryl halides†

**DOI:** 10.1039/d4ra01200c

**Published:** 2024-05-10

**Authors:** Sanaz HajimohamadzadehTorkambour, Masoumeh Jadidi Nejad, Farzane Pazoki, Farzaneh Karimi, Akbar Heydari

**Affiliations:** a Chemistry Department, Tarbiat Modares University P. O. Box 14155-4838 Tehran Iran heydar_a@modares.ac.ir; b Department of Chemistry, Isfahan University of Technology P. O. Box 84156-83111 Isfahan Iran

## Abstract

The utilization of magnetic nanoparticles in the fields of science and technology has gained considerable popularity. Among their various applications, magnetic nanoparticles have been predominantly employed in catalytic processes due to their easy accessibility, recoverability, effective surface properties, thermal stability, and low cost. In this particular study, cyanuric chloride and arginine were utilized to synthesize an arginine-based oligomeric compound (ACT), which was supported on cobalt ferrite, resulting in a green catalyst with high activity and convenient recyclability for the cyanation reaction of aryl halides. The Pd/CoFe_2_O_4_@ACT nanomagnetic catalyst demonstrated excellent performance in the cyanation of various aryl iodides and bromides, yielding favorable reaction outcomes at a temperature of 90 °C within a duration of 3 hours. The synthesized nanoparticles were successfully characterized using various techniques, including FTIR, FE-SEM, EDX/MAP, XRD, TEM, TGA, BET, and ICP-OES. Moreover, the Pd/CoFe_2_O_4_@ACT catalyst exhibited remarkable catalytic activity, maintaining an 88% performance even after five consecutive runs. Analysis of the reused catalyst through SEM and TEM imaging confirmed that there were no significant changes in the morphology or dispersion of the particles. Ultimately, it was demonstrated that the Pd/CoFe_2_O_4_@ACT nanomagnetic catalyst outperformed numerous catalysts previously reported in the literature for the cyanation of aryl halides.

## Abstract 2

The utilization of magnetic nanoparticles in the fields of science and technology has gained considerable popularity.

## Graphical abstract

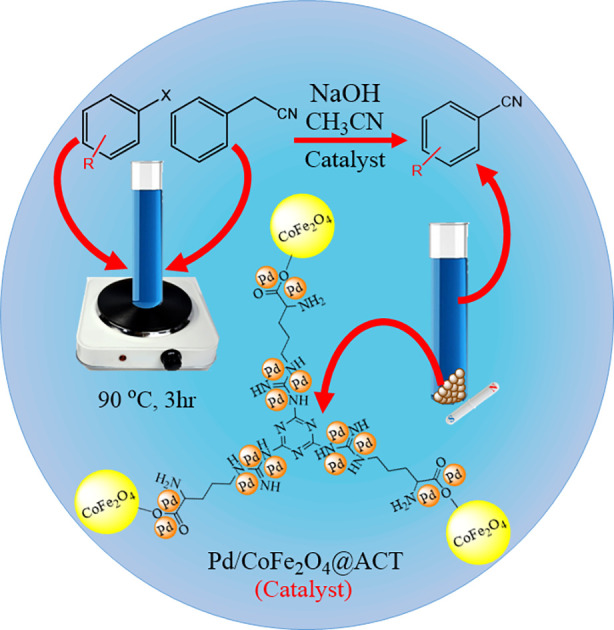

## Introduction

Biocatalysts have played a significant role in scientific research on sustainable chemistry as a source of inspiration. A type of active biocatalytic reaction system is synthesized by functionalized superparamagnetic nanoparticles with biologically active materials, which have high chemical stability, low cost,^1^ low toxicity,^2^ and can be easily separated, recovered, and reused.^3^ In addition, arginine is an important biological molecule due to its wide range of physiological and medicinal functions, which serves as a precursor for synthesizing several biologically significant substances, including amino acids^4^ proteins containing glutamate, polyamines, urea, nitric oxide, and proline.^5^ The present study employed cyanuric chloride and arginine to create an arginine-based oligomer (ACT). In addition, in recent times, nanocatalysts have shown successful performance in various reactions, but their practical application has been limited by the cumbersome process of catalyst recovery through filtration, leading to the loss of solid catalysts.^6^ To overcome this challenge and improve recyclability, magnetic nanocatalysts have been developed. Magnetic nanoparticles have emerged as a strong and high-surface-area support for heterogeneous catalysts. The magnetic properties of these nanocatalysts enable easy separation and recovery using an external magnetic field, which can optimize operational costs and improve the purity of the final product.^7,8^ One of the significant types of magnetic nanoparticles owing to its excellent cubic magneto crystal is cobalt ferrite (CoFe_2_O_4_). In catalysis, ferrites are said to be efficient materials. An organic reaction can be catalyzed by these compounds.^9–11^ Lately, scientists have been focusing on creating nanocatalysts using noble metals, as they exhibit outstanding catalytic performance, possess nanoscale structures, showcase favorable electronic/optical properties, and offer large surface areas.^12,13^ Among these metals, palladium, Ni, Rh, and Ir can be mentioned. Palladium-catalyzed reactions have been used more frequently than Ni-, Rh-, or Ir-catalyzed forms due to the greater tolerance of palladium catalysts to functional groups, selectivity, and high activity.^14–17^ For example, in 2021, 2,4-dichlorophenol (2,4-DCP) was electrochemically dechlorinated using magnetic Pd/CoFe_2_O_4_ catalysts by Xue and Feng.^18^ One of the most important reactions catalyzed by palladium is the cyanation reaction, which involves the introduction of a cyano group onto an aryl halide (Ar-X) and has significant significance in synthetic and industrial chemistry. This is due to the fact that resulting aryl nitriles from the cyanation reaction serve as crucial intermediates that can be further transformed into various functional groups, including carboxylic acids, imines, esters, amines, tetrazoles, aldehydes, and amides. In addition, they are used as versatile organic compounds in chemical and pharmaceutical industries, for example in the synthesis of herbicides, agrochemicals, and dyes.^19^ Recently, the exploration of transition metal-catalyzed cyanation reactions involving aryl halides, utilizing metals such as Ni,^20^ Cu,^21^ and Pd,^22^ has attracted considerable attention. Additionally, a range of protocols employing different cyano sources like NaCN,^23^ KCN,^24^ TMSCN,^22^ Zn(CN)_2_ (ref. 25), and CuCN^26^ have been reported for transition metal-catalyzed cyanation reactions. Nevertheless, most of them are confronted with significant drawbacks, including toxic metal cyanides in stoichiometric amounts and the requirement of harsh reaction conditions. To overcome this problem, several less-toxic metal-free cyanide sources including malononitrile, butyronitrile, acetonitrile, and benzyl cyanide have been reported.^27,28^ Aryl halide cyanations have been reported in various publications utilizing various Pd catalysts, including Pd/C, Pd complexes, and Pd(OAc)_2_.^29–32^ However, the main limitations of these catalysts are their recovery and reuse. In 1973, Takagi *et al.*^33^ introduced the initial application of palladium-catalyzed cyanation, utilizing potassium cyanide in DMF at temperatures ranging from 140 °C to 150 °C for a duration of 2 to 12 hours, specifically targeting bromo- and iodoarenes. Subsequently, in 1986, Chatani and Hanafusa^34^ presented an alternative method for cyanation by employing TMSCN as a cyanide source and Et_3_N as a solvent, focusing on various aryl iodides and using Pd(PPh_3_)_4_ as a catalyst. Later on, alternative sources of cyanide such as Zn(CN)_2_ or CuCN were utilized in palladium-catalyzed reactions. However, both of these sources generate significant amounts of heavy metal waste.^35,36^ In this study, ACT was selected as an integral component of the catalyst because the synthesized ACT structure, in addition to the presence of a high number of NH groups, has triazine rings, and, due to the presence of these rings, ACT is an active site for guest species and a suitable substrate for chemical reactions as a catalyst. 1,3,5-Triazine (and its derivatives) is a very versatile entity, from synthetic (covalent bonds) and supramolecular (coordination, H-bonds, and p-interactions) points of view. Triazine derivatives have proven their great potential in this emerging area of material chemistry, for their π-interaction abilities and their tendency to be involved in intricate H-bond networks. The strong π–π stacking of triazine rings in ACT with aromatic substrates makes reactants more accessible toward Pd active sites, thereby accelerating coupling reactions.^37,38^ As a safe and effective catalyst, Pd/CoFe_2_O_4_@ACT nanoparticles were investigated for the cyanation of aryl halides. It was found that using this catalytic system is an inexpensive, simple, environmentally friendly, and efficient method for cyanation reactions. In addition, the Pd/CoFe_2_O_4_@ACT nanoparticles are magnetic and can be easily separated and recovered by an external magnet and can be used five times without significant activity loss, which demonstrates the practical application of nanocatalysts. The noble metal palladium has also been used, and its superiority in the cyanation reaction of aryl halides compared to other metals has been proven in previous works. Moreover, in this work, using a cyanide source and a less toxic solvent, benzyl cyanide and acetonitrile, respectively, and mild conditions of a temperature of 90 °C and 3 hours, we have obtained products with good performance. Over recent decades, various transition metals have been utilized to catalyze the cyanation reactions of aryls and aryl halides using different cyano sources. Despite many studies on benzonitrile synthesis reactions, we found only one report with a homogeneous palladium catalyst and benzyl cyanide as the cyanide source,^39^ so we were motivated to investigate this reaction under heterogeneous catalytic conditions.

## Materials and methods

First, a detailed review of the equipment used for the present study is presented. Then, a thorough and concise explanation of synthesis processes is provided in the following.

### General remarks

Material requirements were met by purchasing materials from Aldrich (China) and Merck (Germany) companies without any further purification. Thin-layer chromatography was used to monitor the reaction. Using silica-gel 60 F-254 as a matrix, TLC was conducted on glass plates. A Nicolet FT-IR 100 spectrometer was used to obtain infrared (IR) spectra. A Philips X-pert 1710 was used at room temperature to obtain X-ray diffraction (XRD) data. In addition, an energy-dispersive X-ray (EDAX) analysis of the nanoparticles was performed using a TESCAN MIRA III FE-SEM to determine their size and morphology. A Philips EM 208S at 120 kV was used to perform transmission electron microscopy (TEM). In the range of 25–800 °C, a thermal gravimetric analyzer was used to perform thermogravimetric analysis (TGA).

### Synthesis of ACT

**Fig. 1:**
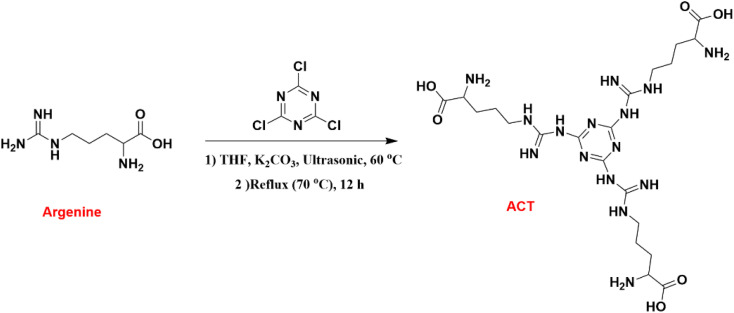
Synthesis of ACT.

In separate processes, 2 mmol of arginine and 1 mmol of cyanuric chloride were dissolved in tetrahydrofuran (5 mL) within a round-bottomed flask under ultrasonication conditions for 10 minutes. The two solutions were then combined, and subsequently, 1 mmol of potassium carbonate was added to the mixture at 60 °C under ultrasonication conditions for 3 hours. The mixture was further stirred at 70 °C for 12 hours. The white solid product was then separated with a centrifuge, eluted with tetrahydrofuran and ethanol, and dried at 80 °C in a vacuum oven. Fig. 1 provides a visual representation of the procedure.

### Synthesis of CoFe_2_O_4_@ACT

After dissolving 1.5 mmol of cobaltous nitrate hexahydrate and 3 mmol of iron(iii) nitrate nonahydrate in 10 mL of deionized water within a round-bottomed flask under ultrasonication for 10 minutes, the two solutions were combined. Subsequently, 0.6 g of ACT was added to the mixture, which was then sonicated using an ultrasonic probe for 30 minutes. To create an alkaline medium with a pH of 12, sodium hydroxide (0.2 M) (20 mL, 0.16 g) was added, and the resulting solution was placed in an autoclave for 24 hours at 120 °C. A magnet was used to separate the end product, which was a light-brown solid, from the medium, and then, deionized water and ethanol were used to purify it. A vacuum oven was used to dry it at 80 °C.

### Synthesis Pd/CoFe_2_O_4_@ACT

First, 0.2 g of CoFe_2_O_4_@ACT was dispersed in deionized water within a round-bottomed flask, and subsequently, 0.11 g of Pd(OAc)_2_ was added to the solution, which was then sonicated for 30 minutes under appropriate conditions. Next, 0.26 g of NaBH_4_ was introduced into the mixture and stirred at an ambient temperature (25 °C) for a period of 24 hours. A magnet was used to isolate the product, which was a dark brown solid, and ethanol and deionized water were used to elute it. Finally, the product was dried at 80 °C in a vacuum oven to obtain the desired end product.

### General procedure for the cyanation of aryl halides

As a solvent, we used 3 mL of acetonitrile to dissolve 1 mmol of aryl halide, 1.5 mmol of benzyl cyanide, 5 mmol of sodium hydroxide, and 0.03 g of catalyst within a test tube, and the mixture was stirred at 90 °C for three hours. The progress of the reaction was monitored using thin-layer chromatography. The mixture was cooled to room temperature after completion of the reaction. An ethyl acetate extraction was then performed on the product after the magnetic catalyst was separated with a magnet. Finally, after the solvent had evaporated under vacuum, the pure product was obtained using column chromatography (ethyl acetate and hexane in a ratio of 1 : 9). Moreover, regarding the characterization of isolated products, it is mentioned that characterizations were based on using ^1^H NMR, ^13^C NMR, and Mass spectroscopy.

## Results and discussion

### Catalyst preparation

**Fig. 2:**
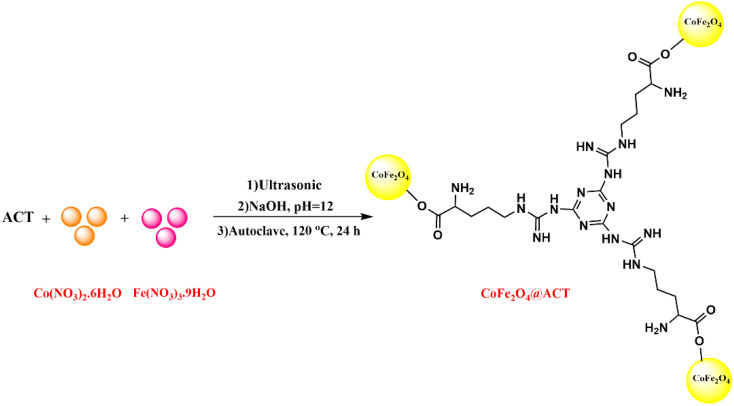
Synthesis of CoFe_2_O_4_@ACT.

**Fig. 3:**
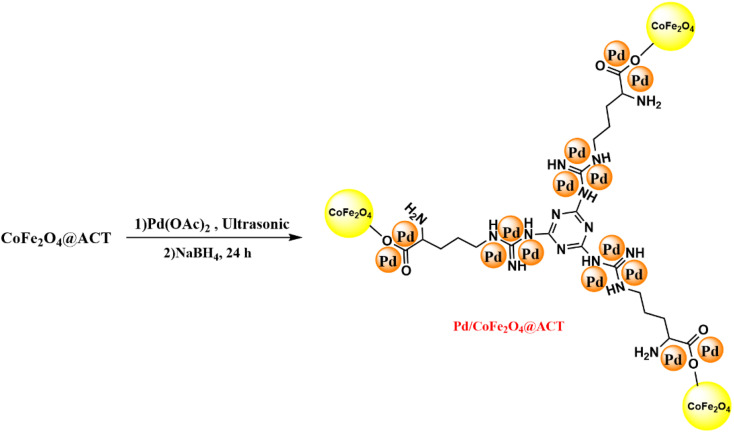
Synthesis of Pd/CoFe_2_O_4_@ACT.

Fig. 1–3 show the preparation process for the Pd/CoFe_2_O_4_@ACT nanocatalyst. Various analyses were conducted on the prepared nanocatalyst, including FTIR, XRD, FE-SEM, TEM, EDX, TGA, ICP, and BET, to determine its structure.

### Characterization of the catalyst

#### FTIR

**Fig. 4:**
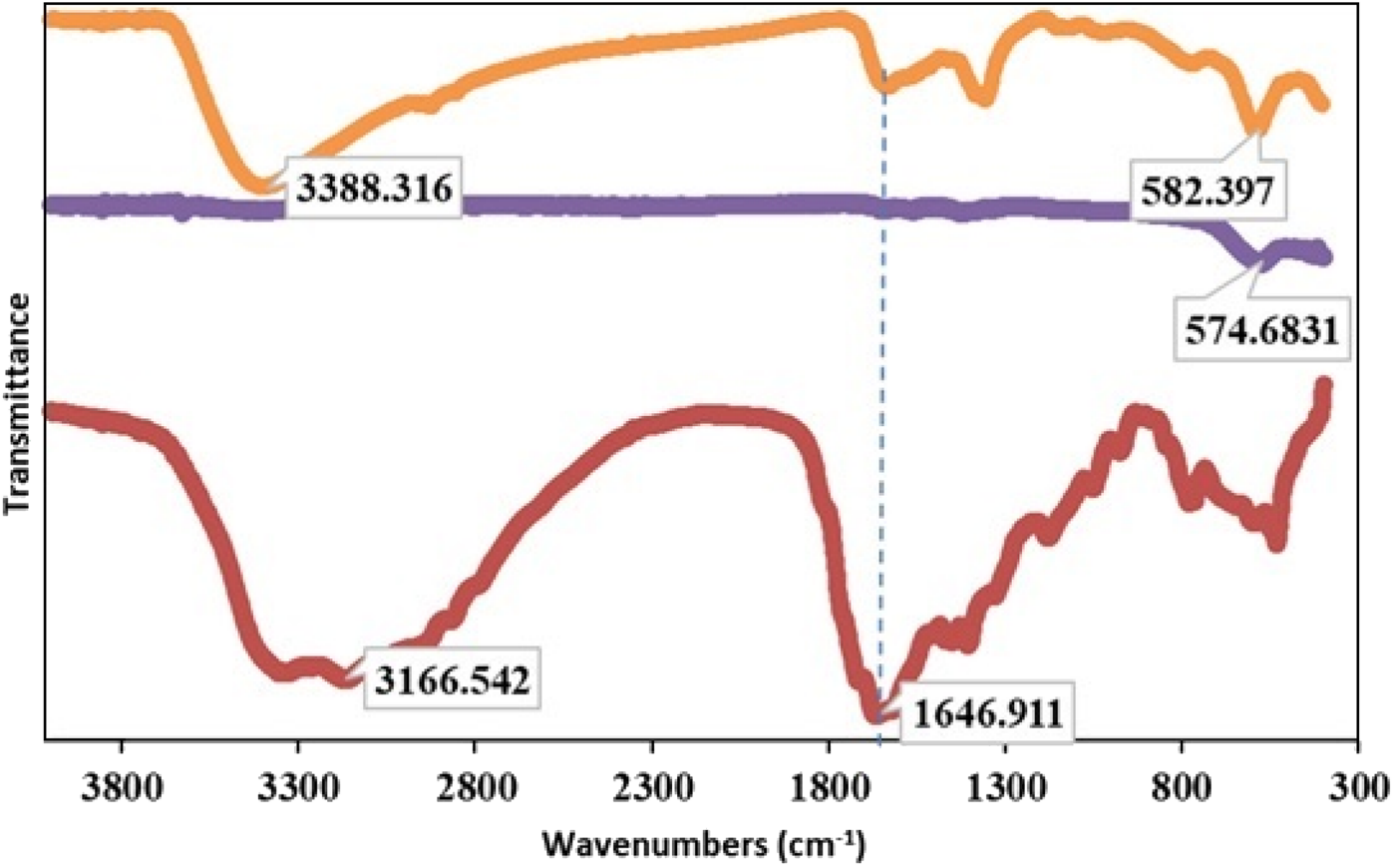
FTIR spectra of Pd/CoFe_2_O_4_@ACT (orange curve), CoFe_2_O_4_ (purple curve), and ACT (red curve).

FTIR spectroscopy provides evidence for the investigation of functional groups and structures. The FTIR spectra of Pd/CoFe_2_O_4_@ACT (orange curve), CoFe_2_O_4_ (purple curve), and ACT (red curve) are shown in Fig. 4. IR bands positioned at about 1714 and 1646.9 cm^−1^ confirm the presence of C=O and N=C in Pd/CoFe_2_O_4_@ACT (orange curve) and ACT (red curve).^40^ The broad absorption bands shown in the region 3000–3300 cm^−1^ correspond to the carboxyl group in ACT (red curve). The bands observed at about 3300 cm^−1^ correspond to stretching vibrations from the adsorbed water and free or adsorbed water on the surface of CoFe_2_O_4_.^41^ M–O (M=metal) stretching bands appear at approximately 578–580 cm^−1^ for Pd/CoFe_2_O_4_@ACT (orange curve) and CoFe_2_O_4_ (purple curve).^42^ This result is in agreement with the formation of the Pd/CoFe_2_O_4_@ACT nanoparticles.

#### TGA

**Fig. 5:**
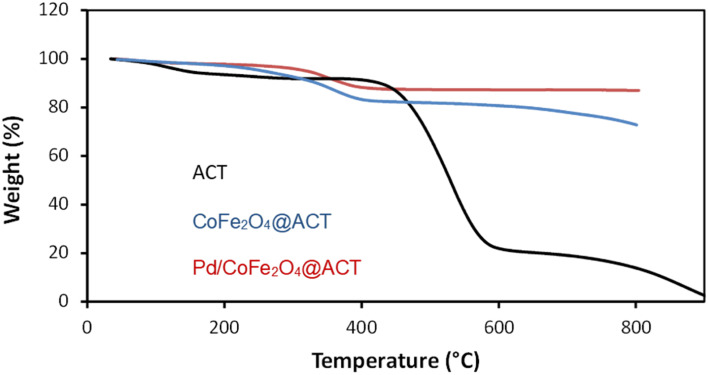
Thermogravimetric analysis of Pd/CoFe_2_O_4_@ACT, CoFe_2_O_4_@ACT, and ACT.

Thermogravimetric analysis (TGA) is a valuable tool to measure the organic content and thermal stability of various substances. As shown in Fig. 5, ACT was decomposed from approximately 220 °C, whereas CoFe_2_O_4_@ACT decomposed from approximately 300 °C. In Pd/CoFe_2_O_4_@ACT, one event is attributed to the loss of adsorbed water molecules at up to 100 °C, while those observed at 400–585 °C were associated with the thermal decomposition of the organic moiety. Moreover, the thermogram establishes that this catalyst was stable up to 400 °C.

#### SEM

**Fig. 6:**
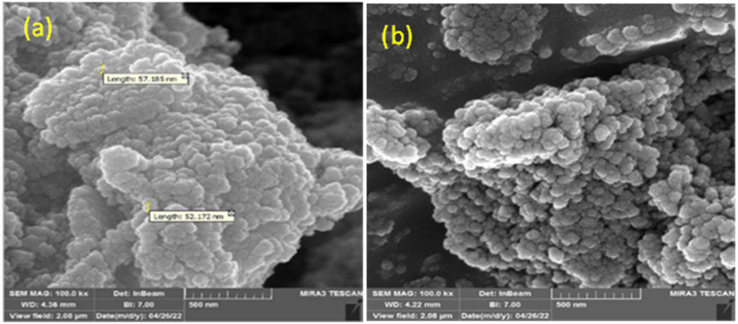
SEM images of Pd/CoFe_2_O_4_@ACT (a and b).

Fig. 6(a and b) illustrates typical SEM images of the Pd/CoFe_2_O_4_@ACT nanoparticles synthesized by the hydrothermal reaction, this analysis being used for investigating the morphology, surface, and size of nanoparticles. Due to the anisotropic growth of crystals on the surface, large bulks appeared on the surface with agglomeration. Moreover, it was observed that the appearance of Pd/CoFe_2_O_4_@ACT is shapeless sphere-like, and the particle size of Pd/CoFe_2_O_4_@ACT is about 52–57 nm.

#### TEM

**Fig. 7:**
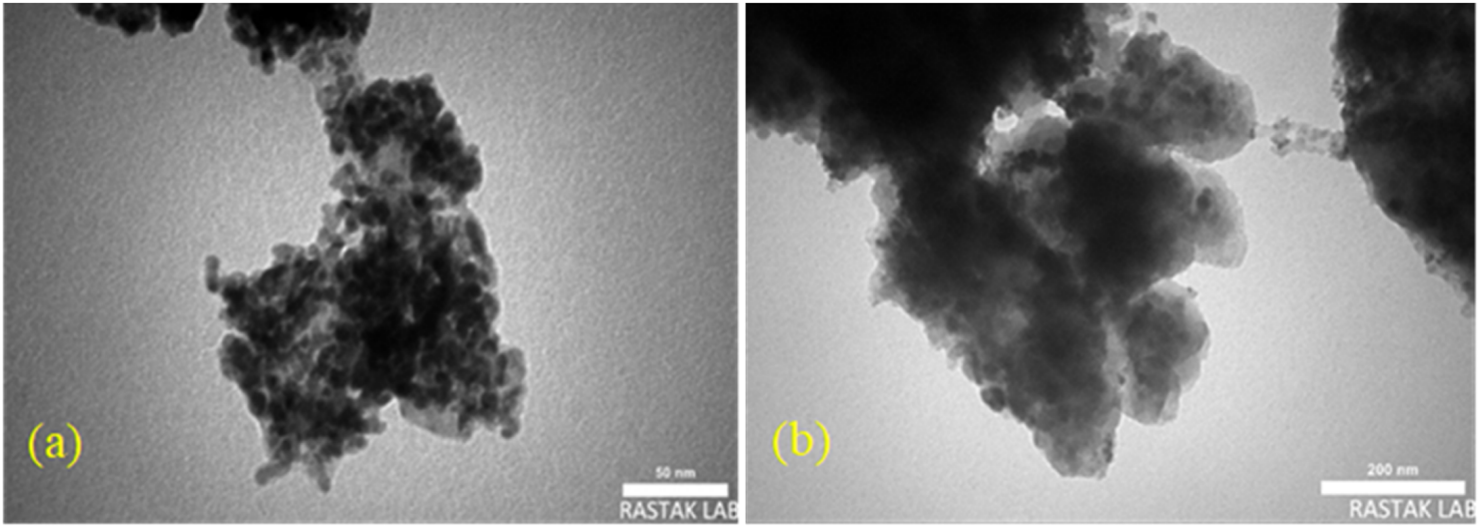
TEM images of Pd/CoFe_2_O_4_@ACT (a and b).

The morphology and particle size of the Pd/CoFe_2_O_4_@ACT photocatalyst were studied from the TEM images of nanoparticles, shown in Fig. 7(a and b). The TEM image indicates that particle size and morphology distribution are uniform for nanoparticles prepared by the hydrothermal treatment method. The size of the smallest nanoparticles was determined at about 50 nm, and the morphology of the particles was sphere-like.

#### EDAX

**Fig. 8:**
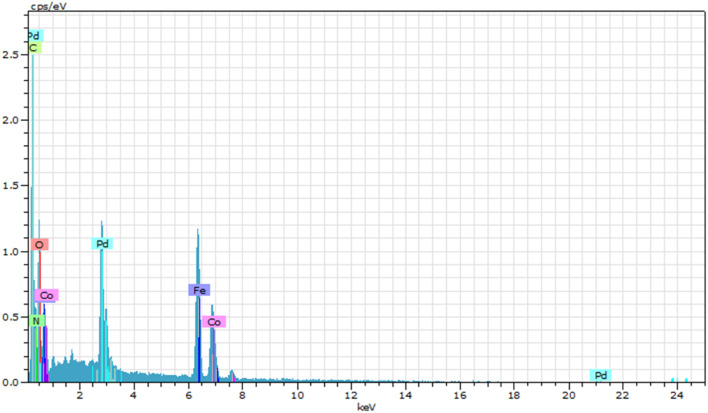
EDX analysis of Pd/CoFe_2_O_4_@ACT.

EDX (or EDS) measurement results indicate the quantitative presence of C, O, N, Fe, Pd, and Co in the samples and, from this analysis, no extra impurities are present in the nanoparticles. EDX analysis of the as-synthesized catalyst is shown in Fig. 8.

The EDX analysis revealed that the Pd/CoFe_2_O_4_@ACT nanocatalyst was predominantly composed of carbon (32.37%), oxygen (26.86%), and nitrogen (20.31%) according to Table 1. Additionally, cobalt, palladium, and iron were present in minor amounts, accounting for 5.71%, 6.24%, and 8.52% of the total composition, respectively.

**Table 1:** Elements in Pd/CoFe_2_O_4_@ACT based on EDX analysis

Element	Weight [%]	Atomic [%]
C	32.37	43.95
O	26.86	27.38
N	20.31	23.65
Fe	8.52	2.49
Pd	6.24	0.96
Co	5.71	1.58

**Fig. 9:**
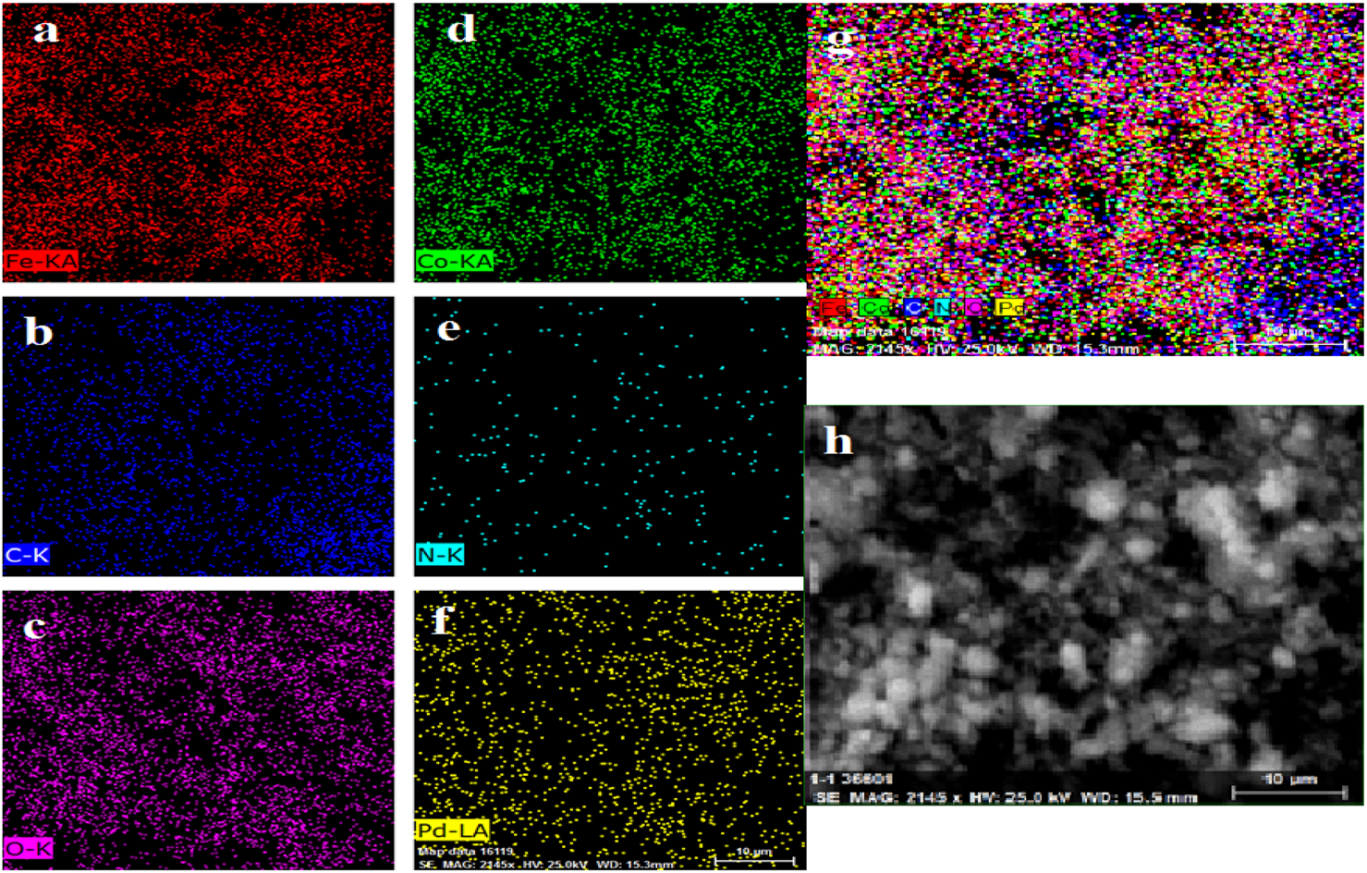
Elemental mapping of (a) Fe-KA, (b) C-K, (c) O-K, (d) Co-KA, (e) N-K, (f) Pd-LA, (g) dispersion of Pd on Pd/CoFe_2_O_4_@ACT, and (h) SEM/EDS image of Pd/CoFe_2_O_4_@ACT.

The images (Fig. 9a–f) are labeled as Fe-KA, C-K, O-K, Co-KA, N-K, and Pd-LA for Pd/CoFe_2_O_4_@ACT that present all the key elements of C, N, O, Fe, and Pd, demonstrated clearly with elemental mapping images (Fig. 9) without the presence of any signature of substituted metals; these also demonstrate the uniform dispersion of Pd on Pd/CoFe_2_O_4_@ACT.

#### BET

**Fig. 10:**
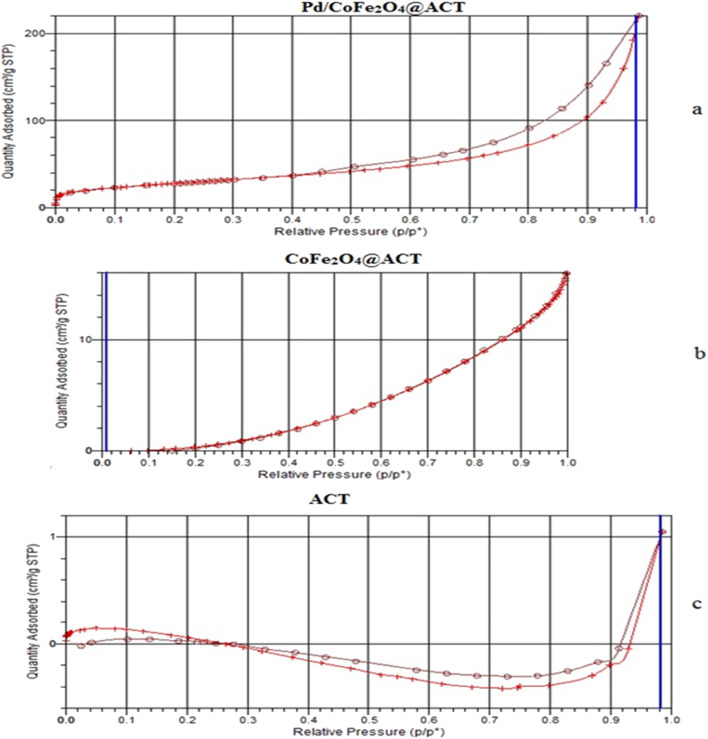
BET results of (a) Pd/CoFe_2_O_4_@ACT, (b) CoFe_2_O_4_@ACT, and (c) ACT.

N_2_ sorption isotherms show the morphological properties of Pd/CoFe_2_O_4_@ACT (curve a), CoFe_2_O_4_@ACT (curve b), and ACT (curve c) in Fig. 10. The surface area of Pd/CoFe_2_O_4_@ACT (curve a), CoFe_2_O_4_@ACT (curve b), and ACT (curve c) was 0.97, 0.67, and 0.55 m^2^ g^−1^, respectively. The average pore diameter was determined using the Barrett–Joyner–Halenda (BJH) method, and it was obtained as 44.78, 50.84, and 105.32 Å, respectively. One of the important properties of nanomaterials is porosity, which is applied for catalytic usage due to the catalytic activity being improved by a high surface area. According to the IPUAC classification, the N_2_ sorption isotherm of Pd/CoFe_2_O_4_@ACT is a type II isotherm, indicative of a nonporous or microporous material.

#### XRD

**Fig. 11:**
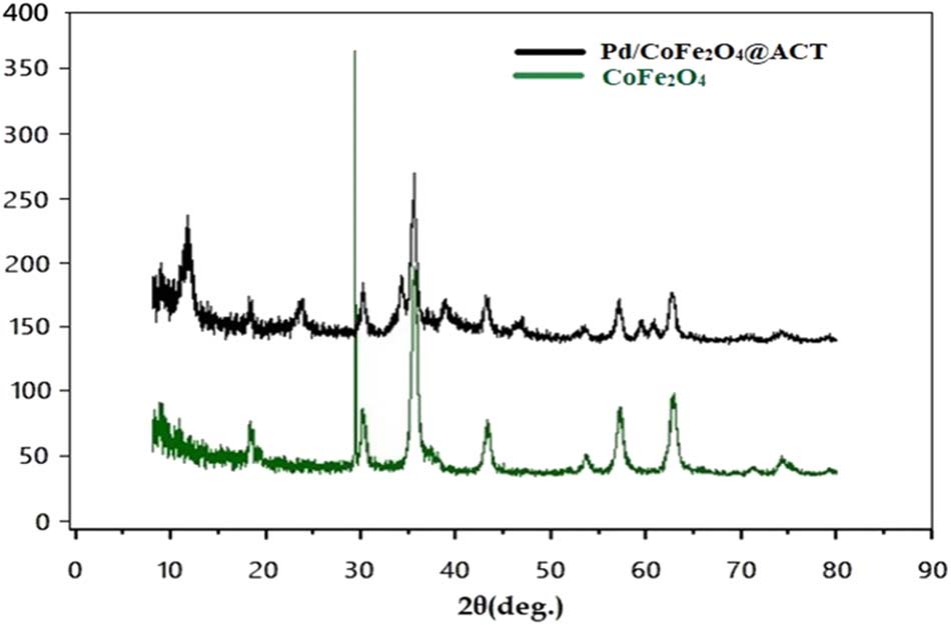
XRD patterns of Pd/CoFe_2_O_4_@ACT and CoFe_2_O_4_.

The X-ray diffraction (XRD) patterns allow structural characterizations of Pd/CoFe_2_O_4_@ACT and CoFe_2_O_4_ using Cu Kα radiation (Fig. 11). The pattern of CoFe_2_O_4_ (green curve) shows characteristic peaks at 19°, 30°, 36°, 37°, 44°, 53°, 56°, and 63°, which represent (1 1 1), (2 2 0), (3 1 1), (2 2 2), (4 0 0), (4 2 2), (5 1 1), and (4 4 0) planes in the nanoparticle crystal structure, respectively. In other words, the XRD pattern confirms the formation of CoFe_2_O_4_. Furthermore, the XRD pattern of Pd/CoFe_2_O_4_@ACT displays peaks at 2*θ* = 40°, 46°, which are attributed to the formation of Pd nanoparticles, and two peaks are detected at 2*θ* of around 11° and 26°, which correspond to the (001) and (100) planes of the ACT ligand.

### Application of the Pd/CoFe_2_O_4_@ACT catalyst in cyanation reaction

**Fig. 12:**
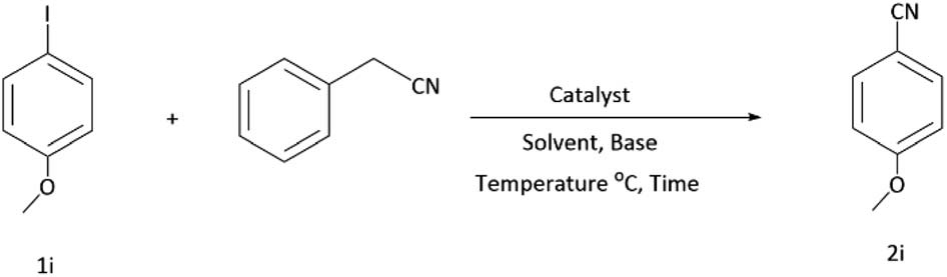
Model reaction for cyanation of aryl halides.

After characterization of the Pd/CoFe_2_O_4_@ACT catalyst, we studied its catalytic performance in the cyanation reaction of aryl halides using benzyl cyanide as the cyanide source (Fig. 12). Initially, we focused our attention on the catalytic activity of the Pd/CoFe_2_O_4_@ACT catalyst, which was comprised of several catalysts (Table 2) in the cyanation reaction. The synthesized Pd/CuFe_2_O_4_@ACT, Pd/NiFe_2_O_4_@ACT, and Pd/CuBi_2_O_4_@ACT nanoparticles (Table 2, entries 2, 3, and 4) were evaluated, which could afford the product in 65%, 50%, and 25% yields, respectively. In contrast, the synthesized Pd/CoFe_2_O_4_@ACT nanocatalyst (Table 2, entry 1) was suitable for this reaction due to its high reaction efficiency with a yield under same conditions of 88%.

**Table 2:** Comparative activity of some selected catalysts toward cyanation reaction^a^

Entry	Catalyst	Yield^b^ (%)
1	Pd/CoFe_2_O_4_@ACT	88
2	Pd/CuFe_2_O_4_@ACT	65
3	Pd/NiFe_2_O_4_@ACT	50
4	Pd/CuBi_2_O_4_@ACT	25

a Reaction conditions: benzyl cyanide (0.175 g, 1.5 mmol), 4-iodoanisole (0.234 g, 1 mmol), K_2_CO_3_ (0.7 g, 5 mmol) (5 mol%), CH_3_CN (5 mL), and catalyst (0.03 g), 8 h, 90 °C (oil bath).

b Isolated yield.

Then, the amount of catalyst was checked for this reaction (Table 3), and 0.03 g of catalyst was found as optimum (Table 3, entry 1). Hence, no change in the reaction yield was observed with the increasing amount of catalyst.

**Table 3:** Pd-catalyzed cyanation using various amounts of catalyst^a^

Entry	Amount of catalyst (g)	Yield^b^ (%)
1	0.03	88
2	0.05	88
3	0.04	88
4	0.02	73
5	0.01	60
6	—	—

a Reaction conditions: benzyl cyanide (0.175 g, 1.5 mmol), 4-iodoanisole (0.234 g, 1 mmol), K_2_CO_3_ (0.7 g, 5 mmol) (5 mol%), CH_3_CN (5 mL), and Pd/CoFe_2_O_4_@ACT, 8 h, 90 °C (oil bath).

b Isolated yield.

As shown in Table 4, the effect of several solvents with different polarities on the cyanation reaction was studied. DMF, DMSO, toluene, and H_2_O were checked, but a trace amount of product was obtained. In the presence of ethanol and THF, the amount of product was improved, and an enhancement of reaction yield was observed when acetonitrile was used as a solvent to 88%.

**Table 4:** Effect of solvent on the reaction yield^a^

Entry	Solvent	Yield^b^ (%)
1	THF	82
2	DMF	—
3	C_4_H_9_NO	20
4	DMSO	—
5	Toluene	—
6	Ethanol	70
7	H_2_O	—
8	CH_3_CN	88

a Reaction conditions: benzyl cyanide (0.175 g, 1.5 mmol), 4-iodoanisole (0.234 g, 1 mmol), K_2_CO_3_ (0.7 g, 5 mmol) (5 mol%), solvent (5 mL), and Pd/CoFe_2_O_4_@ACT (0.03 g), 8 h, 90 °C (oil bath).

b Isolated yield.

Afterward, several bases were tested, and Table 5 summarizes their results. Low reaction yield was observed when the reaction was carried out in the absence of a base (Table 5, entry 4), while the amount of product was enhanced using Cs_2_CO_3_ and NaOAc as a base (Table 5, entries 2 and 1). NaOH was determined as the base of choice for this reaction.

**Table 5:** Pd-catalyzed cyanation using several bases^a^

Entry	Base	Time (h)	Yield^b^ (%)
1	NaOAc	8	88
2	Cs_2_CO_3_	8	90
3	Et_3_N	8	78
4	—	8	74
5	CaCO_3_	8	78
6	NaOH	8	95
7	K_2_CO_3_	8	88

a Reaction conditions: benzyl cyanide (0.175 g, 1.5 mmol), 4-iodoanisole (0.234 g, 1 mmol), base (5 mmol) (5 mol%), CH_3_CN (5 mL), and Pd/CoFe_2_O_4_@ACT (0.03 g), 8 h, 90 °C (oil bath).

b Isolated yield.

In addition, by changing the temperature of the reaction to 90 °C, the amount of product was enhanced to 95% (Table 6, entry 4). Moreover, a higher temperature than 90 °C was checked, and the yield of the reaction did not improve.

**Table 6:** Pd-catalyzed cyanation of aryl halides using several temperatures^a^

Entry	Temperature^c^ (°C)	Time (h)	Yield^b^ (%)
1	100	8	95
2	110	8	95
3	80	8	88
4	90	3	95
5	90	4	95
6	90	5	95
7	90	6	95
8	90	7	95

a Reaction conditions: benzyl cyanide (0.175 g, 1.5 mmol), 4-iodoanisole (0.234 g, 1 mmol), NaOH (0.2 g, 5 mmol) (5 mol%), CH_3_CN (5 mL), and Pd/CoFe_2_O_4_@ACT (0.03 g).

b Isolated yield.

c Oil bath temperature.

Different amounts of benzyl cyanide were tested (Table 7). An amount of 1.5 mmol of benzyl cyanide seemed sufficient to afford a high yield. Thus, the amount of product decreased with an amount of benzyl cyanide lower than 1.5 mmol.

**Table 7:** Pd-catalyzed cyanation of aryl halides using several amounts of benzyl cyanide^a^

Entry	Amount of benzyl cyanide (mmol)	Yield^b^ (%)
1	2	95
2	3	95
3	1.5	95
4	1	89

a Reaction conditions: benzyl cyanide, 4-iodoanisole (0.234 g, 1 mmol), NaOH (0.2 g, 5 mmol) (5 mol%), CH_3_CN (5 mL), and Pd/CoFe_2_O_4_@ACT (0.03 g), 3 h, 90 °C (oil bath).

b Isolated yield.

Afterward, the substrate scope for the cyanation reaction was investigated, and the effect of varying the aryl halide was also explored when reacted with benzyl cyanide (Table 8). Moreover, the scope of the reaction is presented in Table 8. A diverse range of functional groups was analyzed in optimized reaction conditions and compared to electron withdrawing and electron donating groups, such as NO_2_, OCH_3_, OH, Br, and NH_2_. This led to a satisfactory yield of product. Probably, 1-bromo-2-nitrobenzene was tolerated sterically hindered, which afforded a moderate yield (Table 8, entry 11). Furthermore, 1-iodo-4-methoxybenzene, 1-iodo-4-nitrobenzene, and 1-iodo-4-hydroxybenzene underwent the reaction to afford a good yield (Table 8, entries 13, 8, and 9). In addition, a comparison of reaction efficiencies between aryl iodides and aryl bromides reveals the former's superiority, attributed to the weaker C–I bond in contrast to the C–Br bond.^14,27^

**Table 8:** Synthesis of benzonitrile derivatives catalyzed by Pd/CoFe_2_O_4_@ACT^a^

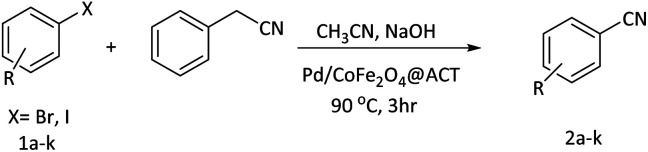			
Entry	Substrate	Product	Yield^b^ (%)
1	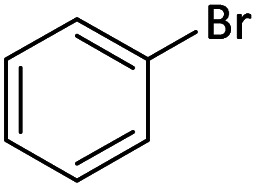	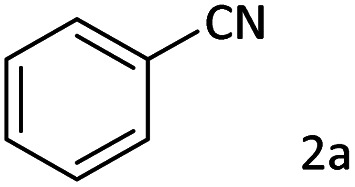	60
2	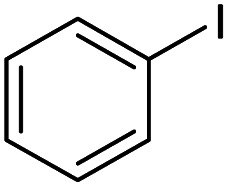	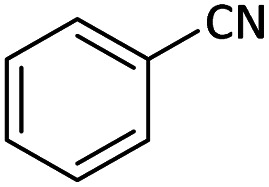	69
3	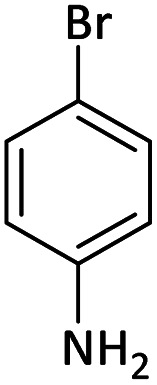	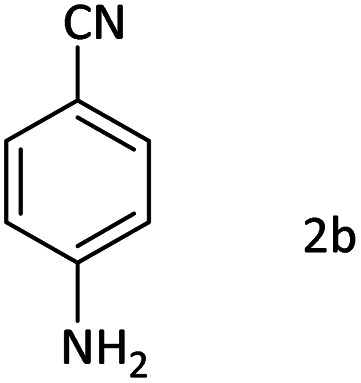	81
4	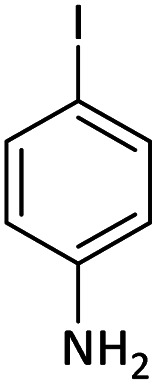	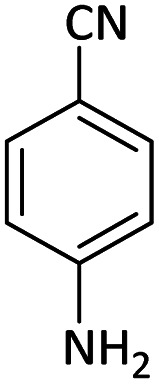	92
5	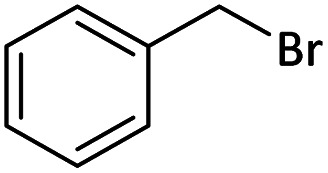	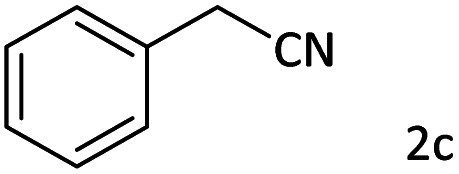	70
6	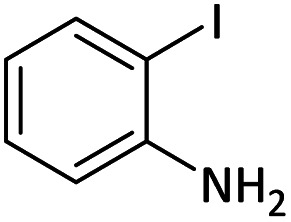	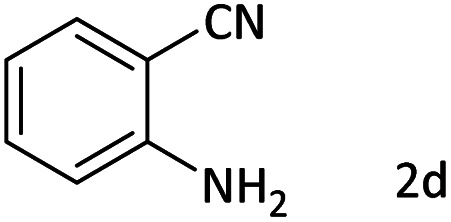	65
7	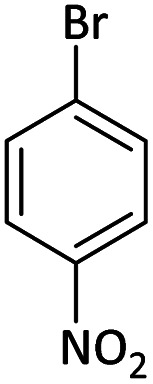	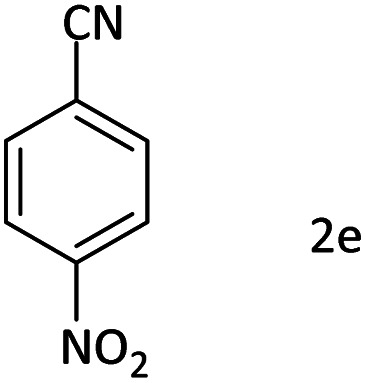	75
8	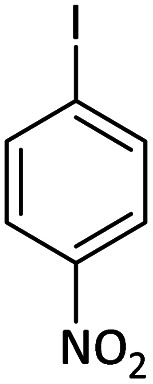	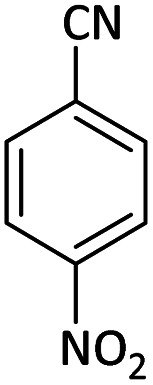	90
9	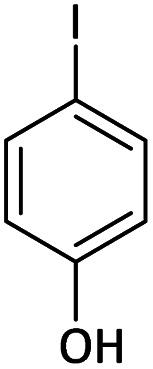	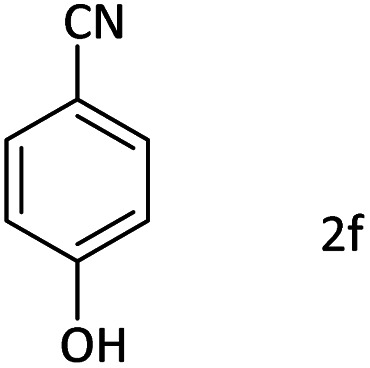	92
10	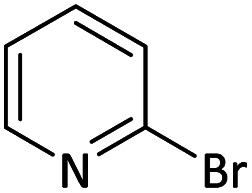	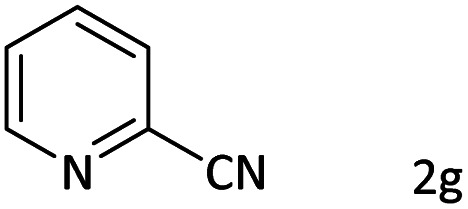	93
11	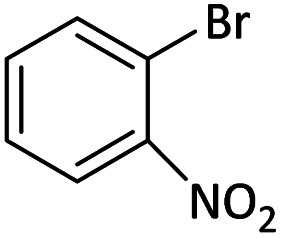	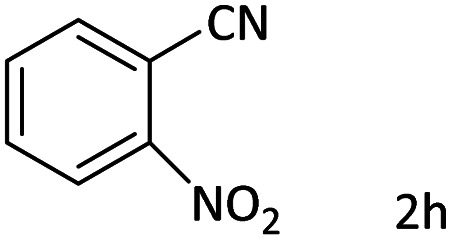	78
12	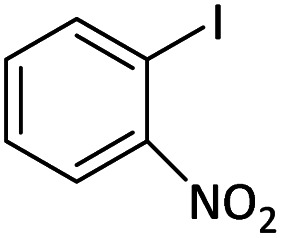	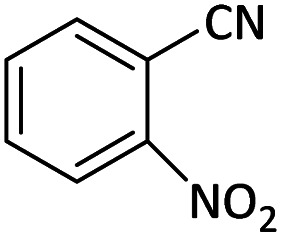	85
13	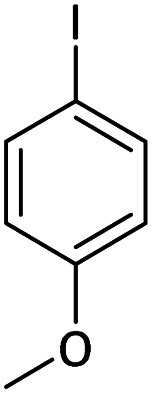	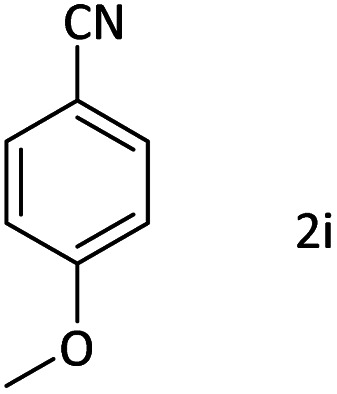	95
14	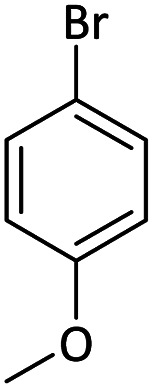	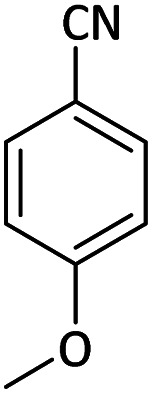	90
15	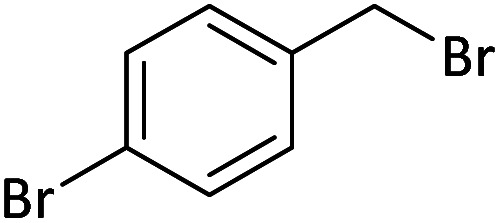	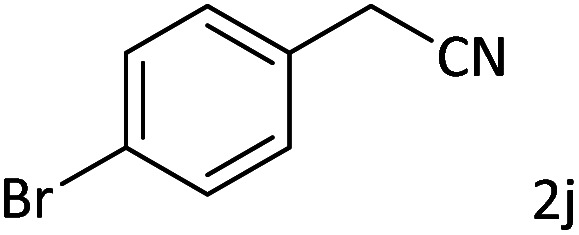	92
16	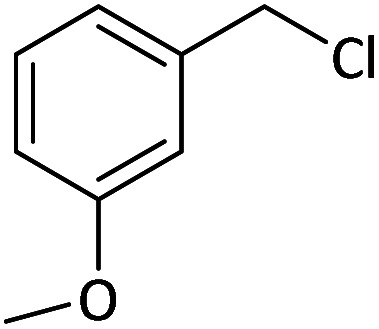	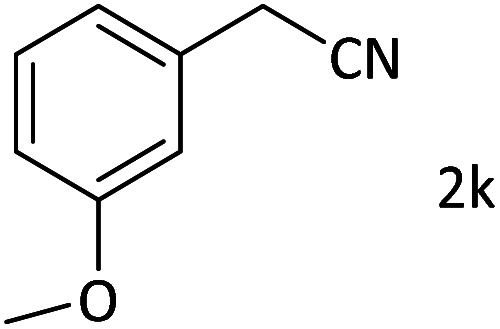	75

a Reaction conditions: benzyl cyanide (0.175 g, 1.5 mmol), 4-iodoanisole (0.234 g, 1 mmol), NaOH (0.2 g, 5 mmol) (5 mol%), CH_3_CN (5 mL), and Pd/CoFe_2_O_4_@ACT (0.03 g), 3 h, 90 °C (oil bath).

b Isolated yield.

Additionally, in the utilization of the Pd/CoFe_2_O_4_@ACT nanoparticles, it was observed that electron-rich or electron-deficient substitutions were not pivotal factors affecting product yields. Furthermore, analysis from Table 8 indicates that aryl halides with O-substitution (2d and 2h) yielded lower results than their counterparts with P-substitution (2b and 2e), suggesting a hindrance effect as a contributing factor.

Further, for the cyanation of aryl halides, Pd/CoFe_2_O_4_@ACT was compared with other reported catalysts, as shown in Table 9. We have developed a catalyst system (Pd/CoFe_2_O_4_@ACT) that has almost the same efficiency as other reported systems in a shorter time and at a lower temperature, which highlights the superiority of our catalyst.

**Table 9:** Comparison of Pd/CoFe_2_O_4_@ACT nanomagnetic catalyst with other reported cyanation catalysts

Entry	Catalyst	Temperature (°C)	Time (h)	Yield^a^ (%)	Recyclability (run)	Reference
1	g-Fe_2_O_3_-Pd-NHC-*n*-butyl-SO_3_Na	90	7	98	6	Omarzehi Chahkamali *et al.*^43^
2	Ni(acac)_2_ (5 mol%), AlCl_3_ (10 mol%), bpy (30 mol%)	145	12	90	—	Yang *et al.*^44^
3	Fe_3_O_4_@SiO_2_-TCT/B_5_-Cu(ii)	100	12	94	5	Karimi *et al.*^27^
4	Zn(OAc)_2_	140	24	86	—	Zhao *et al.*^45^
5	Pd/CoFe_2_O_4_/chitosan hybrid nanocatalyst	120	3	98	5	Baran and Nasrollahzadeh^37^
6	Pd/CoFe_2_O_4_@ACT	90	3	95	5	The present study

a Isolated yield.

### Mechanism

**Fig. 13:**
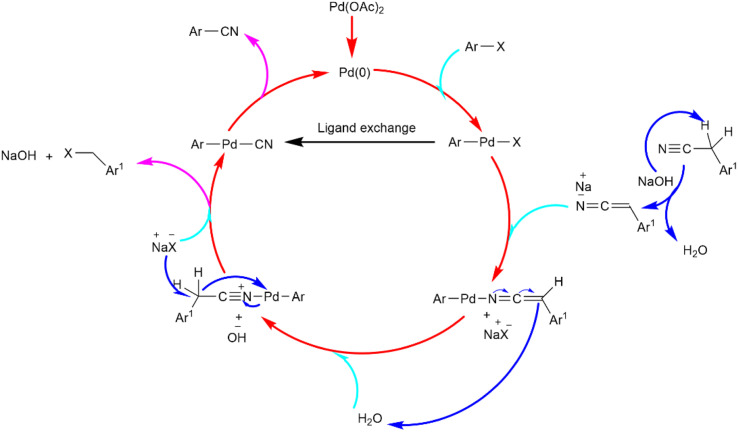
Probable mechanism of the cyanation reaction.

Moreover, an acceptable mechanism for the cyanation reaction is shown in Fig. 13. At first, Pd(0) was produced by reducing Pd(ii). Then, the oxidative addition of Pd(0) to the aryl halide CX bond was carried out by producing a Pd(ii) complex. NaOH attacked benzyl cyanide and removed its hydrogen to produce more strong nucleophiles of benzyl cyanide. Then, the benzyl cyanide nitrogen attacked the complex, and halogen was removed. Next, the halogen attacked and changed the ligand of the complex. Finally, the final product was acquired by reductive elimination.^39^

### Study of reusability and leaching test of the catalyst

**Fig. 14:**
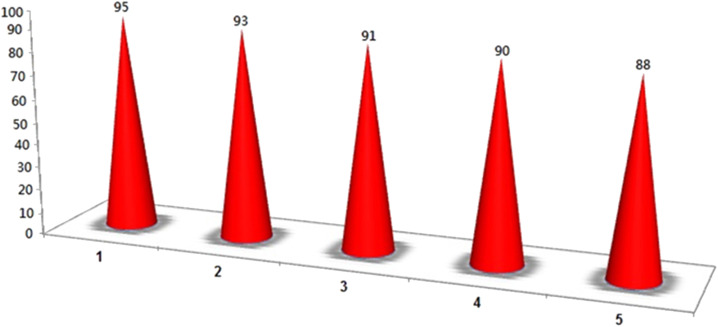
Recycling of the Pd/CoFe_2_O_4_@ACT nanomagnetic catalyst.

Based on the findings of this study, Fig. 14 shows how Pd/CoFe_2_O_4_@ACT was investigated during the cyanation reaction of 1-iodo-4-nitrobenzene in the presence of benzyl cyanide under optimal conditions. After the reaction, Pd/CoFe_2_O_4_@ACT was separated by an external magnet, washed with ethanol, and dried. Then, the catalyst was reused five times. The recovered catalyst was studied in the fifth step with SEM, TEM, and ICP analyses to check the stability of the catalyst under reaction conditions. TEM and SEM analyses after recycling the catalyst five times showed that its particle size, shape, and morphology are not much different from those of the fresh catalyst, which indicates the strength of the catalyst. The recovered nanocatalyst at the fifth stage (0.38 mmol g^−1^) did not exhibit significant palladium leaching when compared to the original catalyst, according to ICP analysis. In another study, when the yield was 58%, the catalyst was magnetically separated, so the reaction could run without it. After the scheduled time, no further progress in the reaction was observed, which means that there was no catalyst washout.

**Fig. 15:**
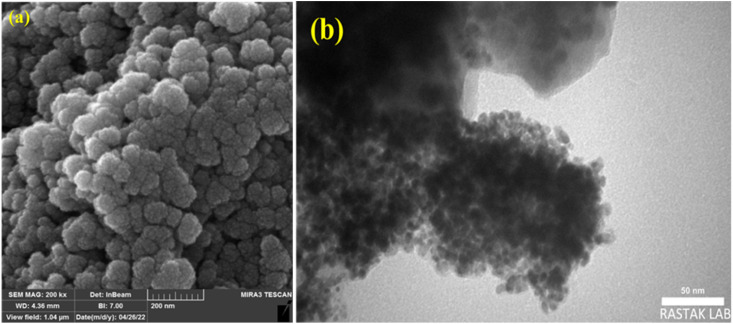
(a) SEM and (b) TEM analysis of the catalyst after the 5th cycle.

Additionally, according to Fig. 15, the SEM and TEM images of the catalyst after being used 5 times show no significant changes in the particle size, shape, and morphology compared to the fresh catalyst.

## Conclusion

In summary, Pd/CoFe_2_O_4_@ACT as a nanomagnetic catalyst was successfully designed and synthesized. For the synthesis of this nanomagnetic and green catalyst, cyanuric chloride, and arginine were immobilized on CoFe_2_O_4_. The nanocatalyst was investigated using FTIR, XRD, BET, SEM, TGA, TEM, ICP-OES, and EDX/MAP. Characterization studies showed that the particle size of the synthesized magnetic nanoparticles (Pd/CoFe_2_O_4_@ACT) is about 52–57 nm. Moreover, these nanoparticles were applied as a green and heterogeneous catalyst for the cyanation reaction of aryl halides using benzyl cyanide as a source of cyanide. The desired products were obtained in a short period of time, at a low temperature, and with a high yield. The main advantages of this method are good yield, simple work-up, stability of the catalyst, and recyclability of the catalyst for five cycles without significant palladium leaching. Furthermore, the Pd/CoFe_2_O_4_@ACT nanomagnetic catalyst performed better than several previously reported catalysts for cyanation of aryl halides.

## Data and code availability

The data that support the findings of this study are available in the supplementary material† of this article.

## Author contributions

Conceptualization: Sanaz HajimohamadzadehTorkambour, Masoumeh Jadidi Nejad; methodology: Sanaz HajimohamadzadehTorkambour, Masoumeh Jadidi Nejad; formal analysis and investigation: Sanaz HajimohamadzadehTorkambour; writing – original draft preparation: Farzane Pazoki, Sanaz HajimohamadzadehTorkambour; writing – review and editing: Farzaneh Karimi, Sanaz HajimohamadzadehTorkambour; funding acquisition: Akbar Heydari; resources: Akbar Heydari; supervision: Akbar Heydari.

## Conflicts of interest

The authors declare that they have no known competing financial interests or personal relationships that could have appeared to influence the work reported in this paper.

## Supplementary Material

RA-014-D4RA01200C-s001
