# Supplementary material for: Synthesis and characterization of a green and recyclable arginine-based palladium/CoFe2O4 nanomagnetic catalyst for efficient cyanation of aryl halides
Source: RSC Adv. 2024 May 10;14(20):14139–51. doi: 10.1039/d4ra01200c (PMC11085038; doi:10.1039/d4ra01200c)
Supplement: RA-014-D4RA01200C-s001 [file RA-014-D4RA01200C-s001.pdf]

Electronic Supplementary Material (ESI) for RSC Advances.
This journal is © The Royal Society of Chemistry 2024

# Synthesis and Characterization of a Green and Recyclable Arginine-based Palladium/$CoFe_2O_4$ Nanomagnetic Catalyst for Efficient Cyanation of Aryl Halides

Sanaz HajimohamadzadehTorkambour[a], Masoumeh Jadidi Nejad[b], Farzane Pazoki[a], Farzaneh Karimi[a], and Akbar Heydari*[a]

[a]*Chemistry Department, Tarbiat Modares University, P.O. Box 14155-4838, Tehran, Iran.*

[b]*Department of Chemistry, Isfahan University of Technology, P.O. Box 84156-83111, Isfahan, Iran.*

**E-mail: heydar_a@modares.ac.ir; Fax: +98-21-82883455; Tel: +98-21-82883444.*

**Table of contents**

| | |
|---|---|
| 1. **Characterization of aryl nitrile derivatives** | **S1** |
| 2. **$^1$H NMR and $^{13}$CNMR spectra of aryl nitrile derivatives** | **S4** |
| 3. **References** | **S21** |

## Characterization of aryl nitrile derivatives:

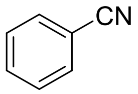

Benzonitrile (2a). Colorless oil; $^1$H NMR (499 MHz, DMSO-$d_6$) δ 7.81 (d, J = 7.6 Hz, 2H), 7.72-7.69 (m, 1H), 7.56 (t, J = 7.6 Hz, 2H); $^{13}$C NMR (126 MHz, DMSO-$d_6$) δ 132.62, 131.15, 128.77, 118.57, 112.64. HRMS (ESI): Calcd for $C_7H_5N$ $(M + H)^+$ 103.04, found 103.09.

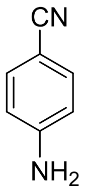

4-Aminobenzonitrile (2b). White solid, mp 83-85 $^o$C; $^1$H NMR (499 MHz, DMSO-$d_6$) δ 7.38 (d, J = 8.1 Hz, 2H), 6.58 (d, J = 8.2 Hz, 2H), 6.12 (s, 2H); $^{13}$C NMR (126 MHz, DMSO-$d_6$) δ 151.00, 133.12, 118.44, 114.66, 98.60. HRMS (ESI): Calcd for $C_7H_6N_2$ $(M + H)^+$ 118.05, found 118.11.

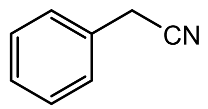


2-Phenylacetonitrile (2c). Colorless oil; $^{1}$H NMR (499 MHz, DMSO-$d_6$) δ 7.39-7.31 (m, 2H), 7.31-7.25 (m, 3H), 3.71 (s, 2H); $^{13}$C NMR (126 MHz, DMSO-$d_6$) δ 129.8, 129.0, 127.9, 127.8, 117.8, 23.4. HRMS (ESI): Calcd for $C_8H_7N$ (M +H) $^{+}$ 117.06, found 117.11.

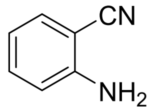


2-Aminobenzonitrile (2d). Beige-brown solid, mp 49-50 $^{o}$ C; $^{1}$H NMR (499 MHz, DMSO-$d_6$) δ 7.35 (d, J = 7.9 Hz, 1H), 7.28 (t, J = 7.5 Hz, 1H), 6.76 (d, J = 8.5 Hz, 1H), 6.57 (t, J = 7.5 Hz, 1H), 5.98 (s, 2H); $^{13}$C NMR (126 MHz, DMSO-$d_6$) δ 151.10, 133.59, 13.95, 118.01, 116.75, 115.05, 94.31. HRMS (ESI): Calcd for $C_7H_6N_2$ (M +H) $^{+}$ 118.05, found 118.08.

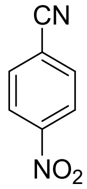


4-Nitrobenzonitrile (2e). Yellow solid, mp 147-149 $^{o}$ C; $^{1}$H NMR (499 MHz, DMSO-$d_6$) δ 8.37 (d, J = 8.3 Hz, 2H), 8.16 (d, J = 8.3 Hz, 2H); $^{13}$C NMR (126 MHz, DMSO-$d_6$) δ 15.01, 133.80, 123.92, 117.93, 117.07. HRMS (ESI): Calcd for $C_7H_4N_2O_2$ (M +H) $^{+}$ 148.03, found 148.08.

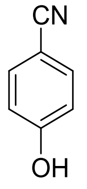


4-Hydroxybenzonitrile (2f). White solid, mp 110-112 $^{o}$ C; $^{1}$H NMR (499 MHz, DMSO-$d_6$) δ 10.54 (s, 1H), 7.55 (d, J = 8.2 Hz, 2H), 6.85 (d, J = 8.3 Hz, 2H); $^{13}$C NMR (126 MHz, DMSO-$d_6$) δ 161.80, 134.22, 118.56, 116.27, 102.33. HRMS (ESI): Calcd for $C_7H_5NO$ (M +H) $^{+}$ 119.04, found 119.18.

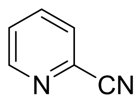


Picolinonitrile (2g). White solid, mp 28-30 $^{o}$ C; $^{1}$H NMR (499 MHz, DMSO-$d_6$) δ 8.75 (d, J = 4.8 Hz, 1H), 8.07-8.01 (m, 2H), 7.73 (t, J = 6.1 Hz, 1H); $^{13}$C NMR (126 MHz, DMSO-$d_6$) δ

151.37, 138.00, 133.98, 128.20, 127.66,117.32. HRMS (ESI): Calcd for $C_6H_4N_2$ (M +H) $^+$ 104.04, found 104.12.

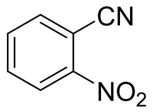

2-Nitrobenzonitrile (2h). Yellow solid, mp 109-110 ° C; $^1$H NMR (499 MHz, DMSO-$d_6$) δ 8.42-8.35 (m, 1H), 8.17 (dd, J = 5.7, 3.4 Hz, 1H), 7.98 (dd, J = 6.0, 3.4 Hz, 2H); $^{13}$C NMR (126 MHz, DMSO-$d_6$) δ 149.39, 135.51, 134.36, 133.83, 124.14, 115.13,107.96. HRMS (ESI): Calcd for $C_7H_4N_2O_2$ (M +H) $^+$ 148.03, found 148.11.

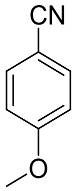

4-Methoxybenzonitrile (2i). White solid, mp 60-61 ° C; $^1$H NMR (499 MHz, DMSO-$d_6$) δ 7.76 (d, J = 8.6 Hz, 2H), 7.09 (d, J = 8.6 Hz, 2H), 3.83 (s, 3H); $^{13}$C NMR (126 MHz, DMSO-$d_6$) δ 163.10, 133.88, 118.55, 114.22, 104.08, 55.32. HRMS (ESI): Calcd for $C_{13}H_{13}N_2O$ (M +H) $^+$ 133.05, found 133.09.

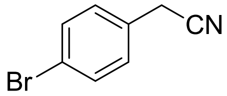

2-(4-bromophenyl)acetonitrile (2j). White solid, mp 48-50 ° C; $^1$H NMR (499 MHz, $CDCl_3$) δ 7.54-7.47 (m, 2H), 7.21 (d, J = 8.5 Hz, 2H), 3.71 (s, 2H); $^{13}$C NMR (126 MHz, DMSO-$d_6$) δ 132.3, 129.6, 128.9, 122.2, 117.3, 23.2. HRMS (ESI): Calcd for $C_8H_6BrN$ (M +H) $^+$ 194.97, found 195.03.

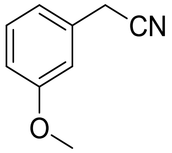

2-(3-methoxyphenyl)acetonitrile (2k). Yellow oil; $^1$H NMR (499 MHz, $CDCl_3$) δ 7.30-7.24 (m, 1H), 6.90-6.84 (m, 3H), 3.80 (s, 3H), 3.71 (s, 2H); $^{13}$C NMR (126 MHz, DMSO-$d_6$) δ 160.26, 131.40, 130.32, 120.28, 117.89, 113.74, 113.70, 55.47, 23.76. HRMS (ESI): Calcd for $C_9H_9NO$ (M +H) $^+$ 147.07, found 147.13.

**$^{1}$H NMR and $^{13}$CNMR spectra of aryl nitrile derivatives:**

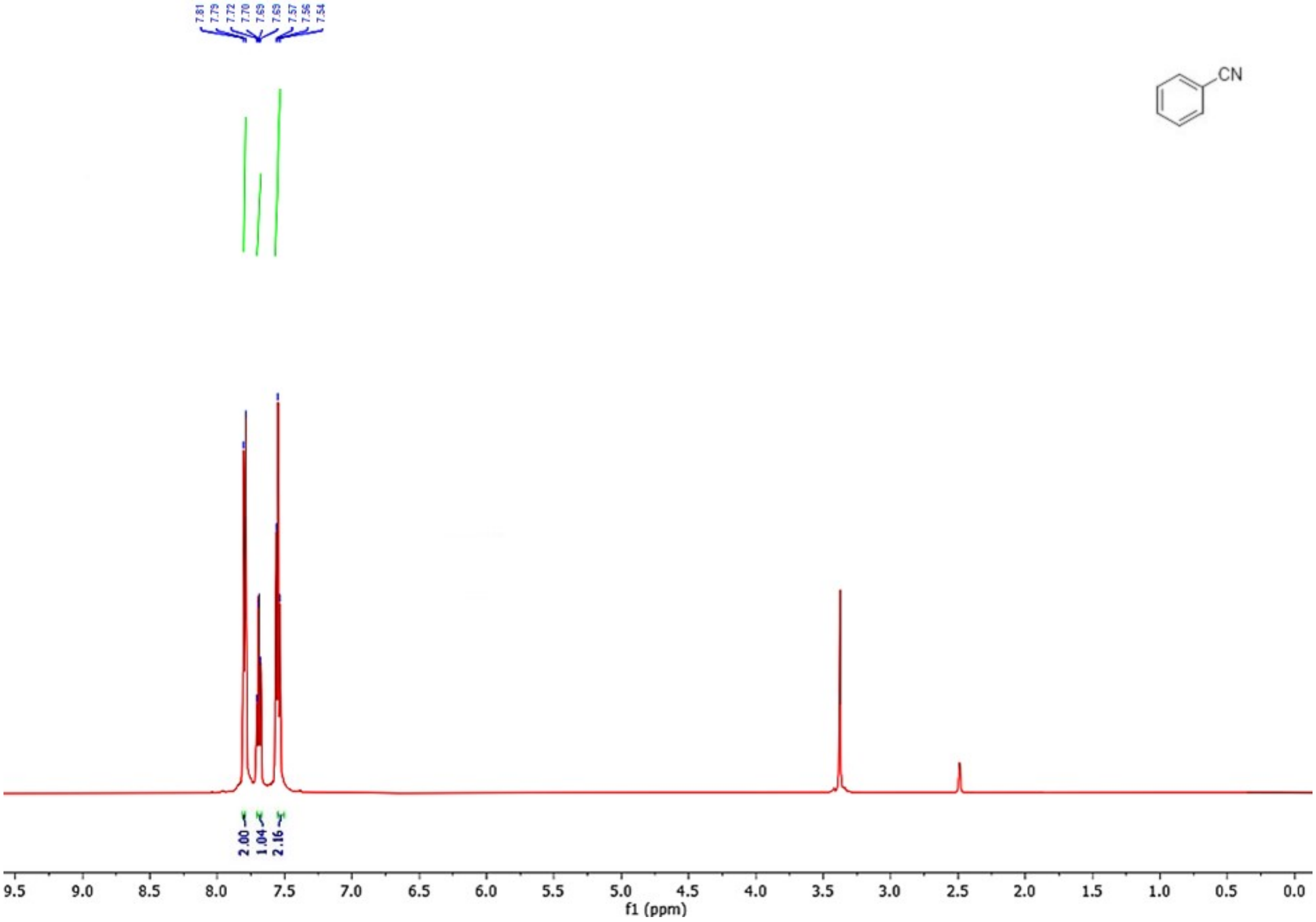

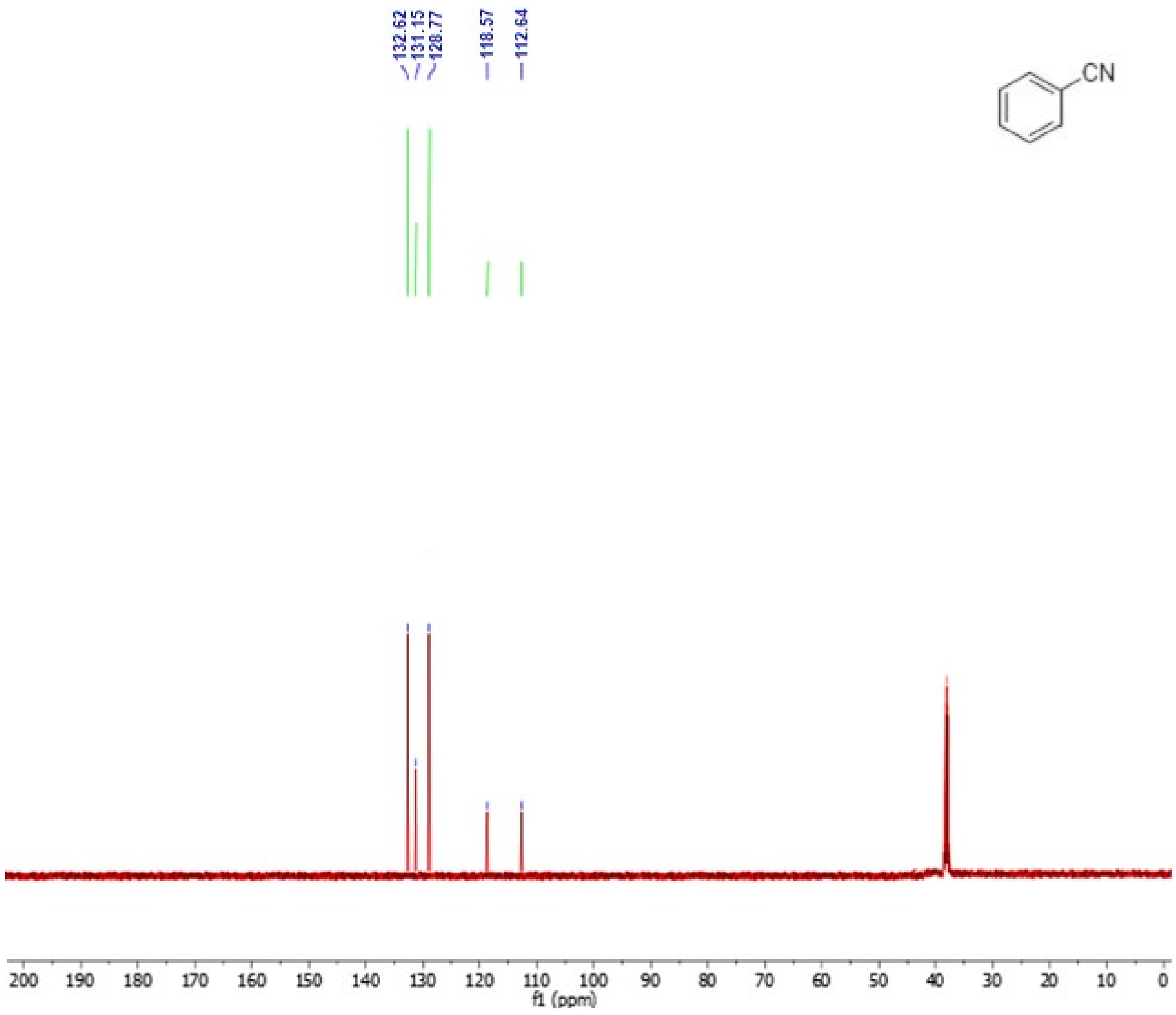
132.62
131.15
128.77
118.57
112.64
CN
200 190 180 170 160 150 140 130 120 110 100 90 80 70 60 50 40 30 20 10 0
f1 (ppm)

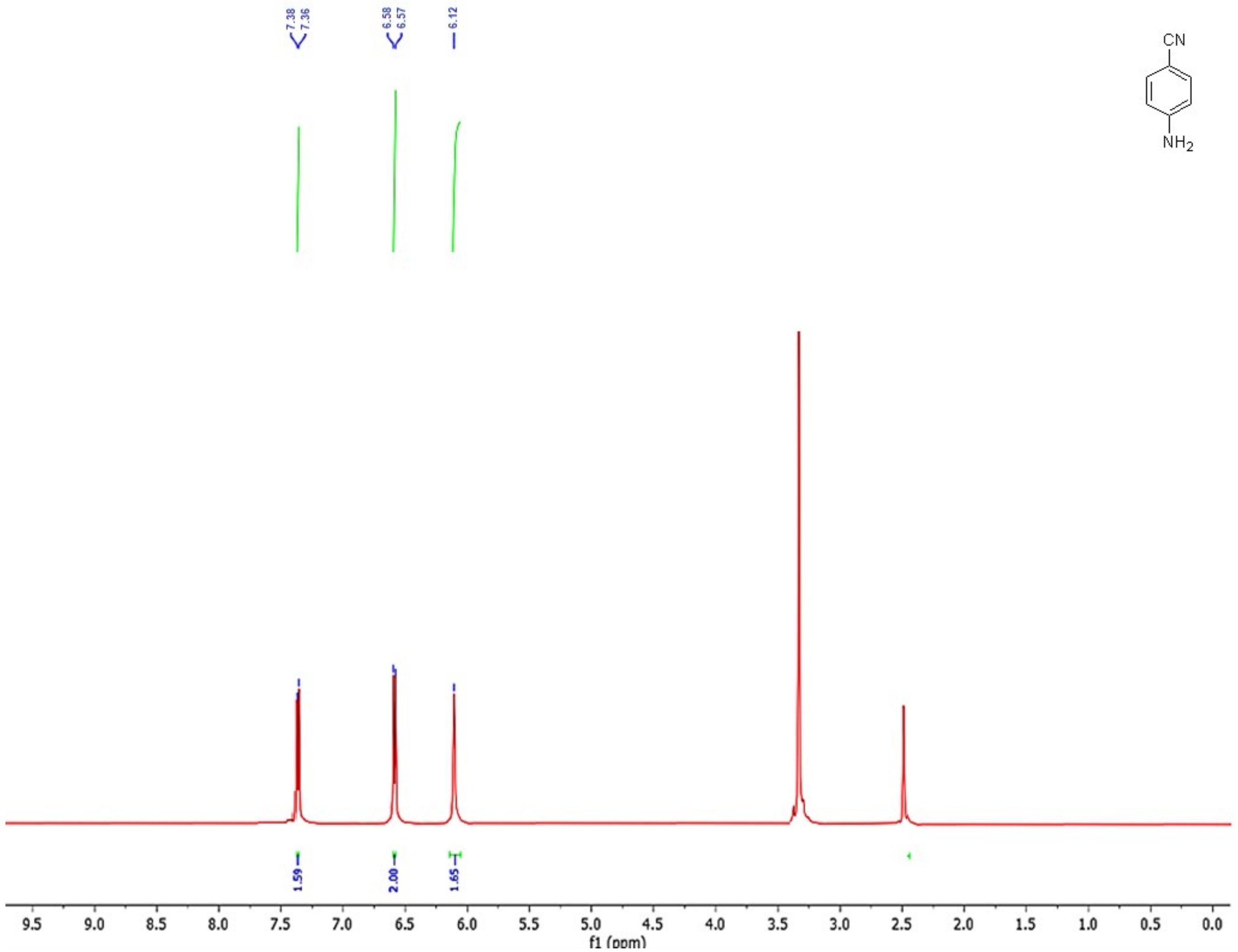

7.38
7.36
6.58
6.57
6.12
CN
NH2
1.59
2.00
1.65
9.5 9.0 8.5 8.0 7.5 7.0 6.5 6.0 5.5 5.0 4.5 4.0 3.5 3.0 2.5 2.0 1.5 1.0 0.5 0.0
f1 (ppm)

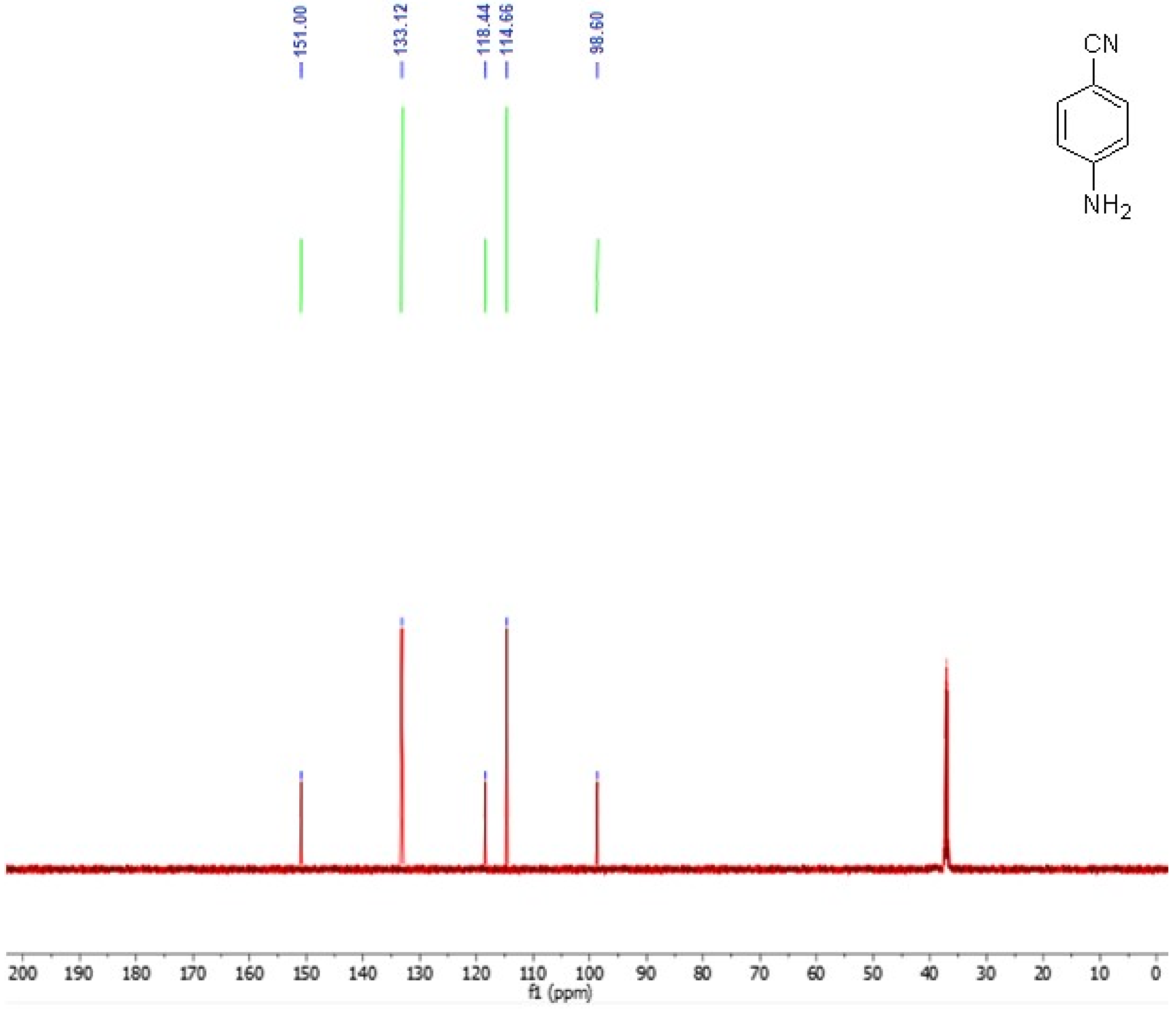
151.00
133.12
118.44
114.66
98.60
CN
NH2
200 190 180 170 160 150 140 130 120 110 100 90 80 70 60 50 40 30 20 10 0
f1 (ppm)

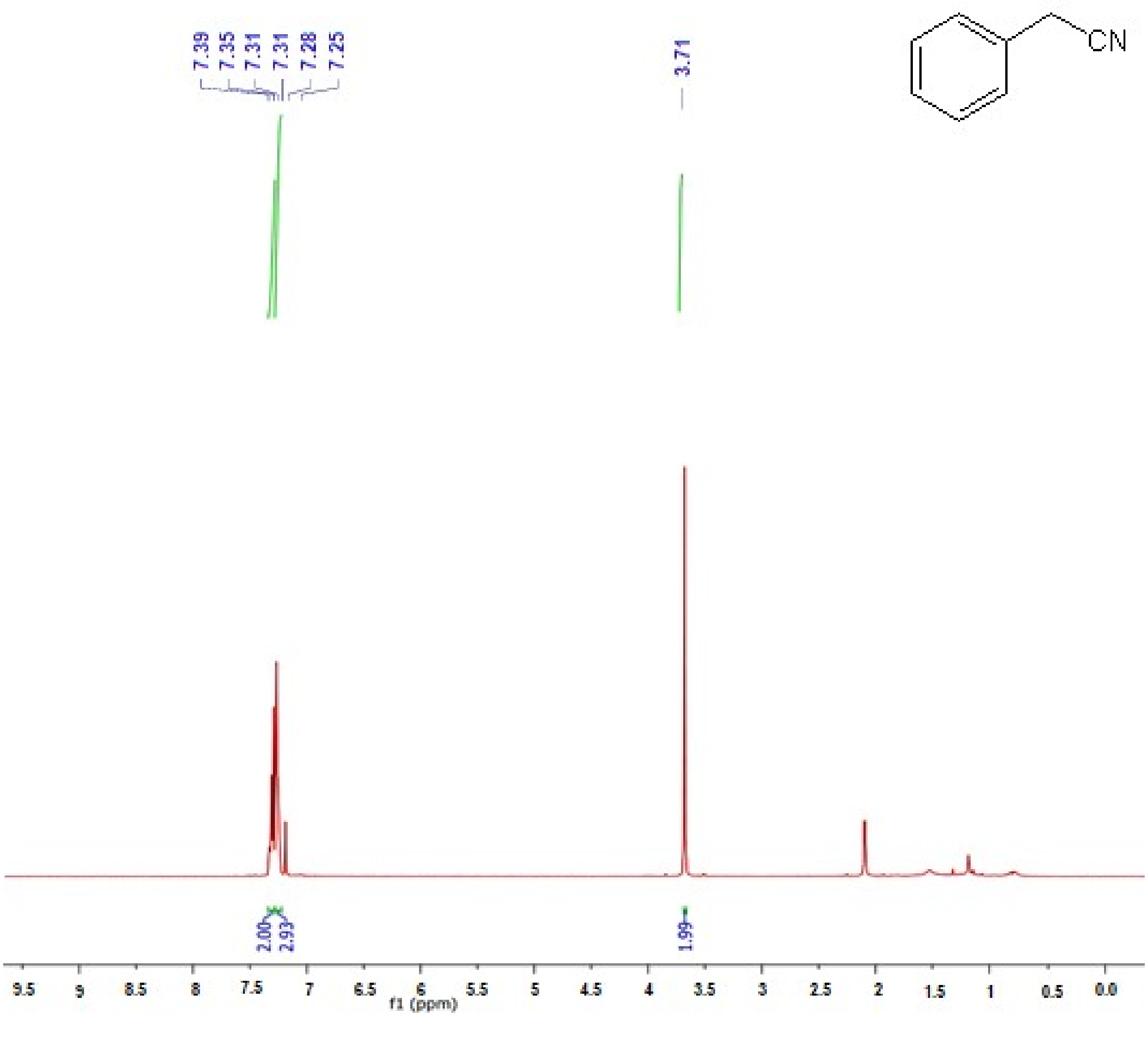

7.39
7.35
7.31
7.31
7.28
7.25
3.71
CN
2.00
2.93
1.99
9.5 9 8.5 8 7.5 7 6.5 6 5.5 5 4.5 4 3.5 3 2.5 2 1.5 1 0.5 0.0
f1 (ppm)

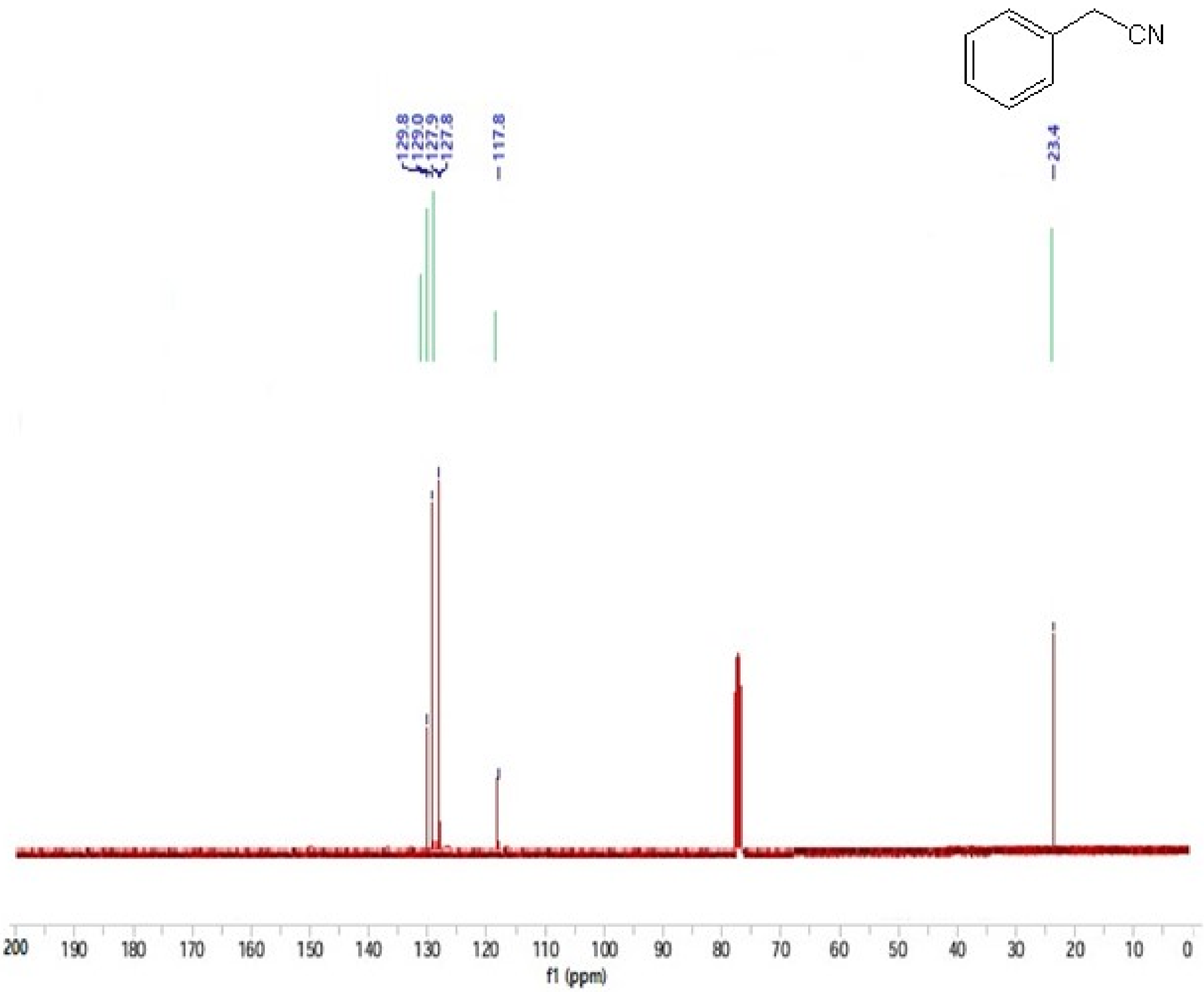
CN
129.8
129.0
127.9
127.8
117.8
23.4
200
190
180
170
160
150
140
130
120
110
100
90
80
70
60
50
40
30
20
10
0
f1 (ppm)

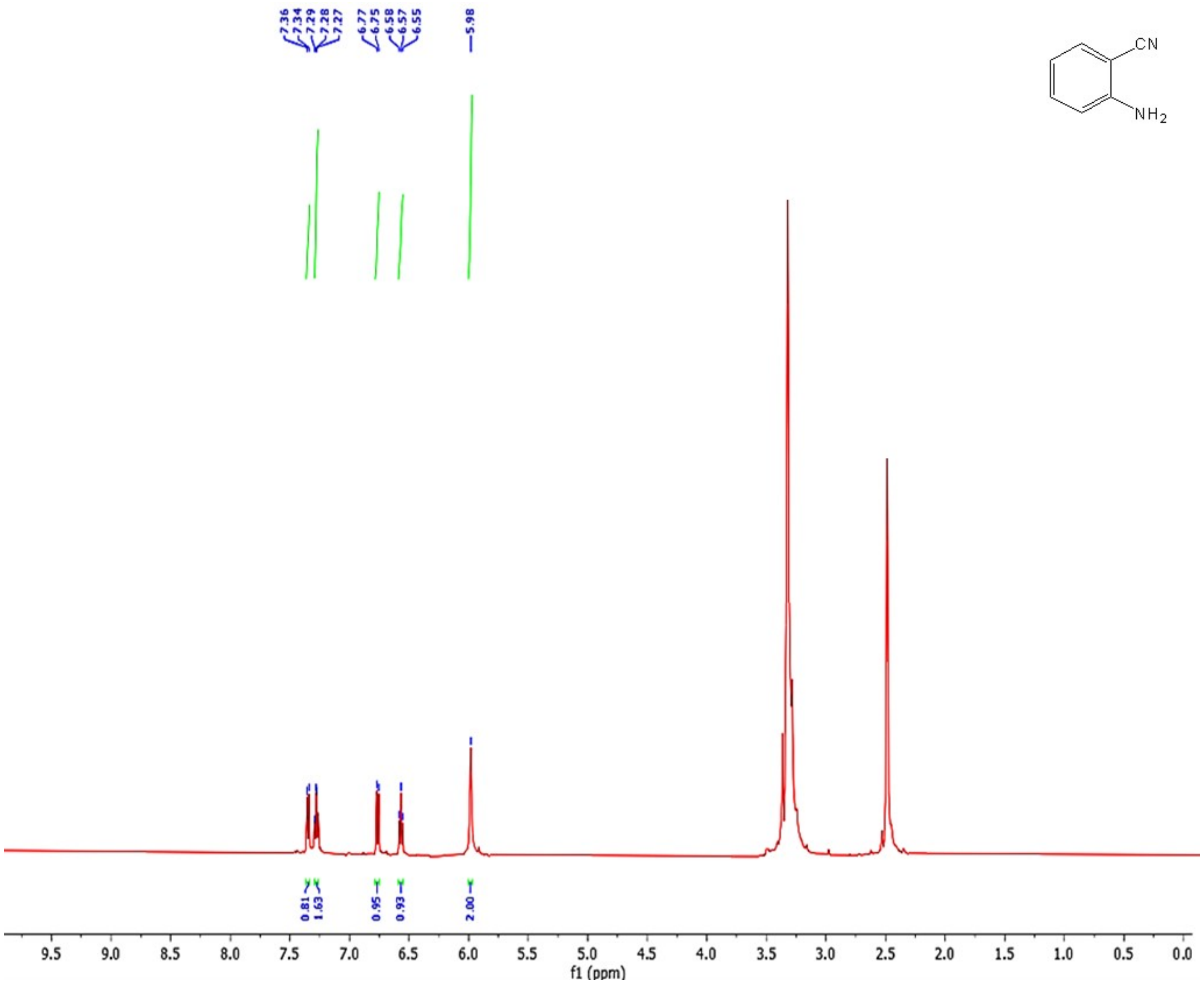
7.36
7.34
7.29
7.28
7.27
6.77
6.75
6.58
6.57
6.55
5.98
CN
NH2
0.81
1.63
0.95
0.93
2.00
9.5 9.0 8.5 8.0 7.5 7.0 6.5 6.0 5.5 5.0 4.5 4.0 3.5 3.0 2.5 2.0 1.5 1.0 0.5 0.0
f1 (ppm)

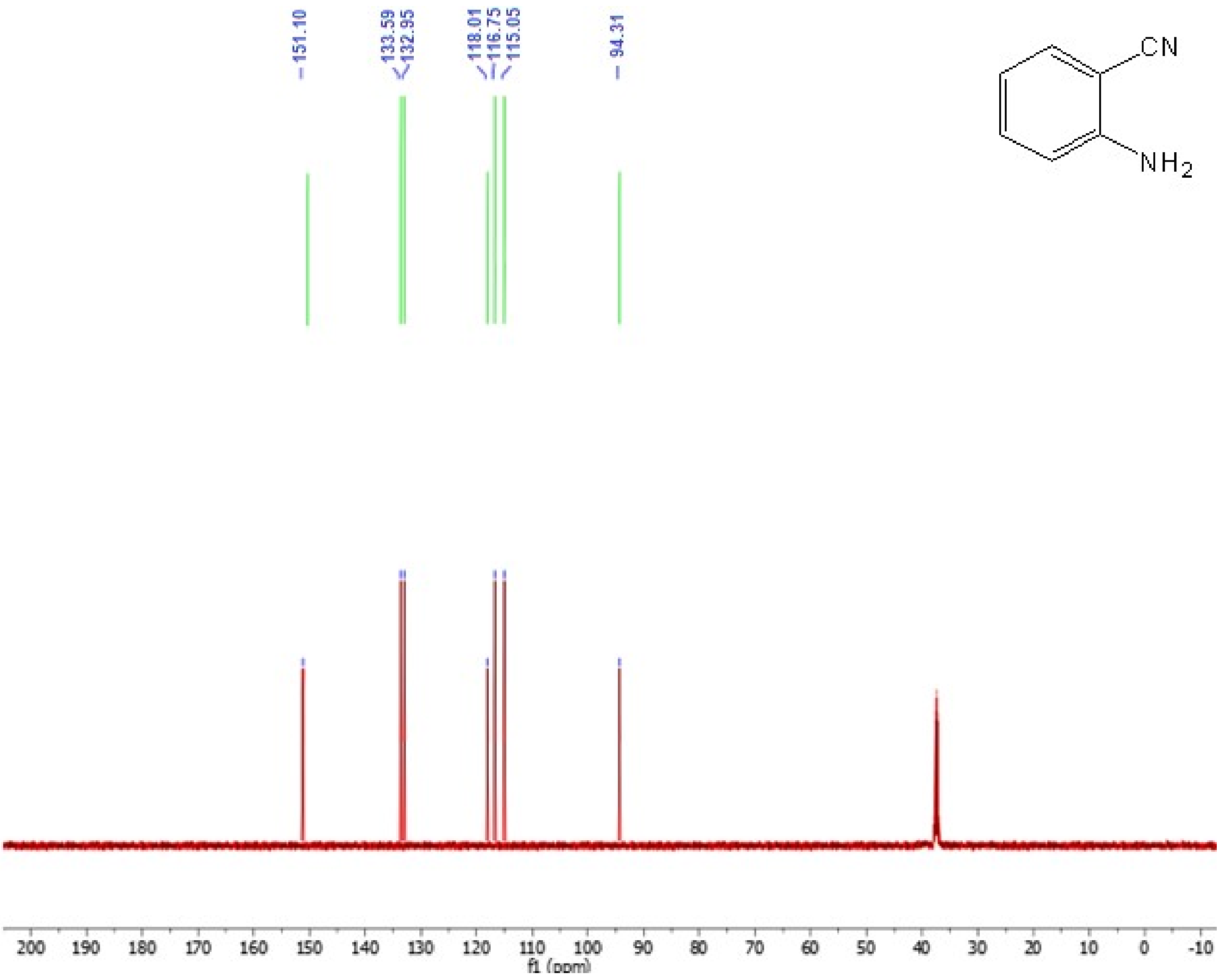
151.10
133.59
132.95
118.01
116.75
115.05
94.31
200 190 180 170 160 150 140 130 120 110 100 90 80 70 60 50 40 30 20 10 0 -10
f1 (ppm)
CN
NH2

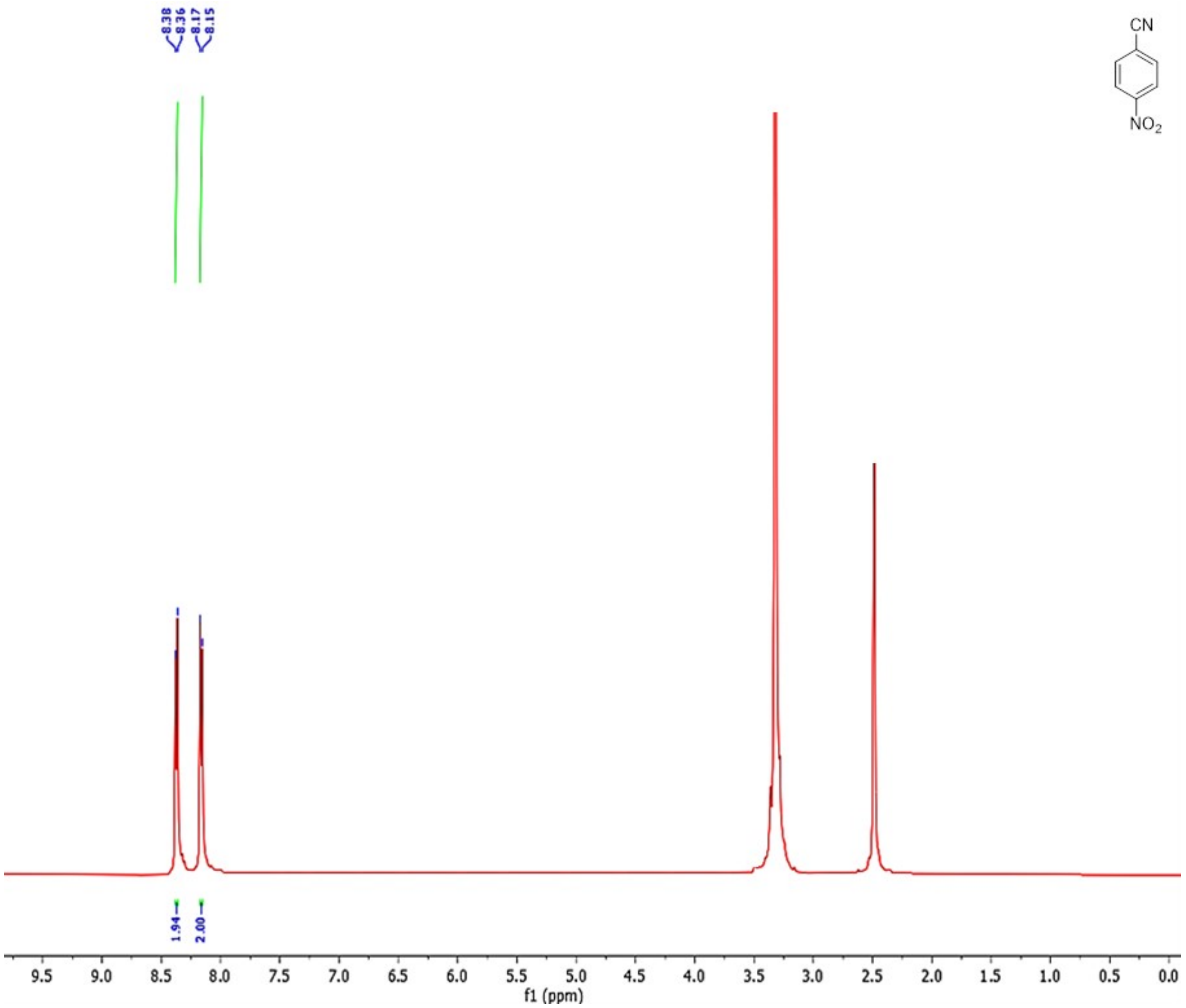
8.38
8.36
8.17
8.15
1.94
2.00
9.5 9.0 8.5 8.0 7.5 7.0 6.5 6.0 5.5 5.0 4.5 4.0 3.5 3.0 2.5 2.0 1.5 1.0 0.5 0.0
f1 (ppm)
CN
NO2

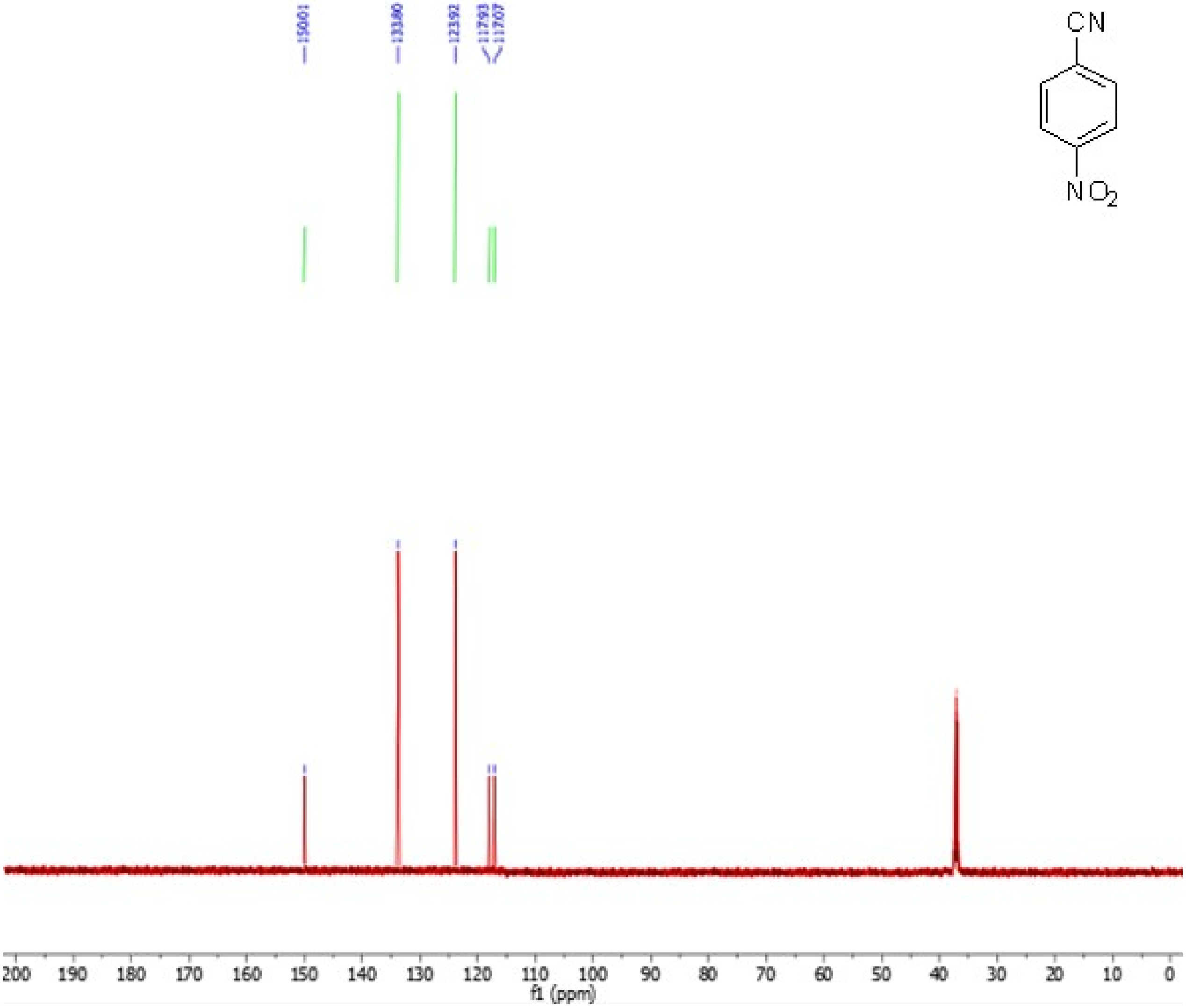
150.01
133.80
123.92
117.93
117.07
CN
NO2
200 190 180 170 160 150 140 130 120 110 100 90 80 70 60 50 40 30 20 10 0
f1 (ppm)

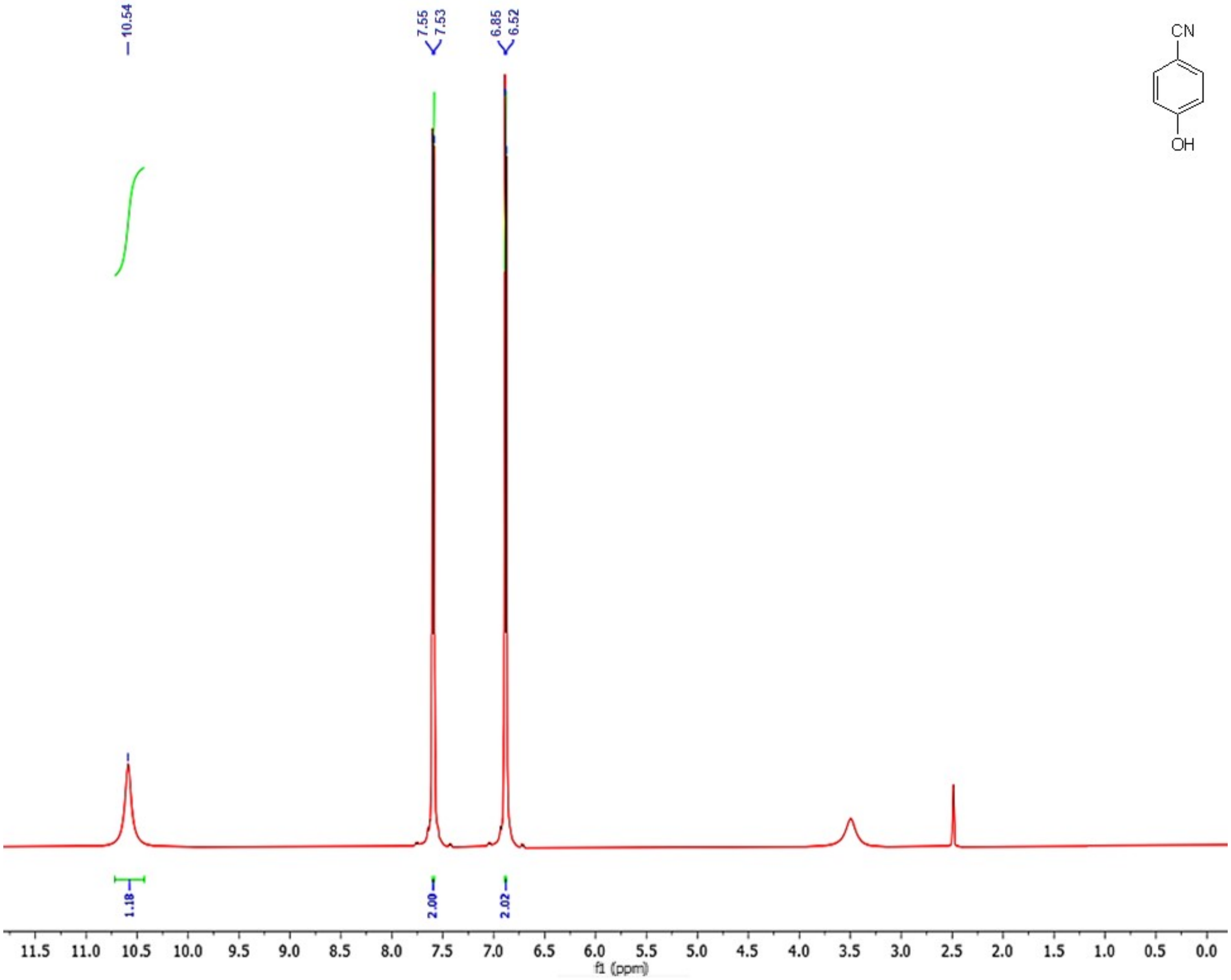

10.54
7.55
7.53
6.85
6.52
CN
OH
1.18
2.00
2.02
11.5 11.0 10.5 10.0 9.5 9.0 8.5 8.0 7.5 7.0 6.5 6.0 5.5 5.0 4.5 4.0 3.5 3.0 2.5 2.0 1.5 1.0 0.5 0.0
f1 (ppm)

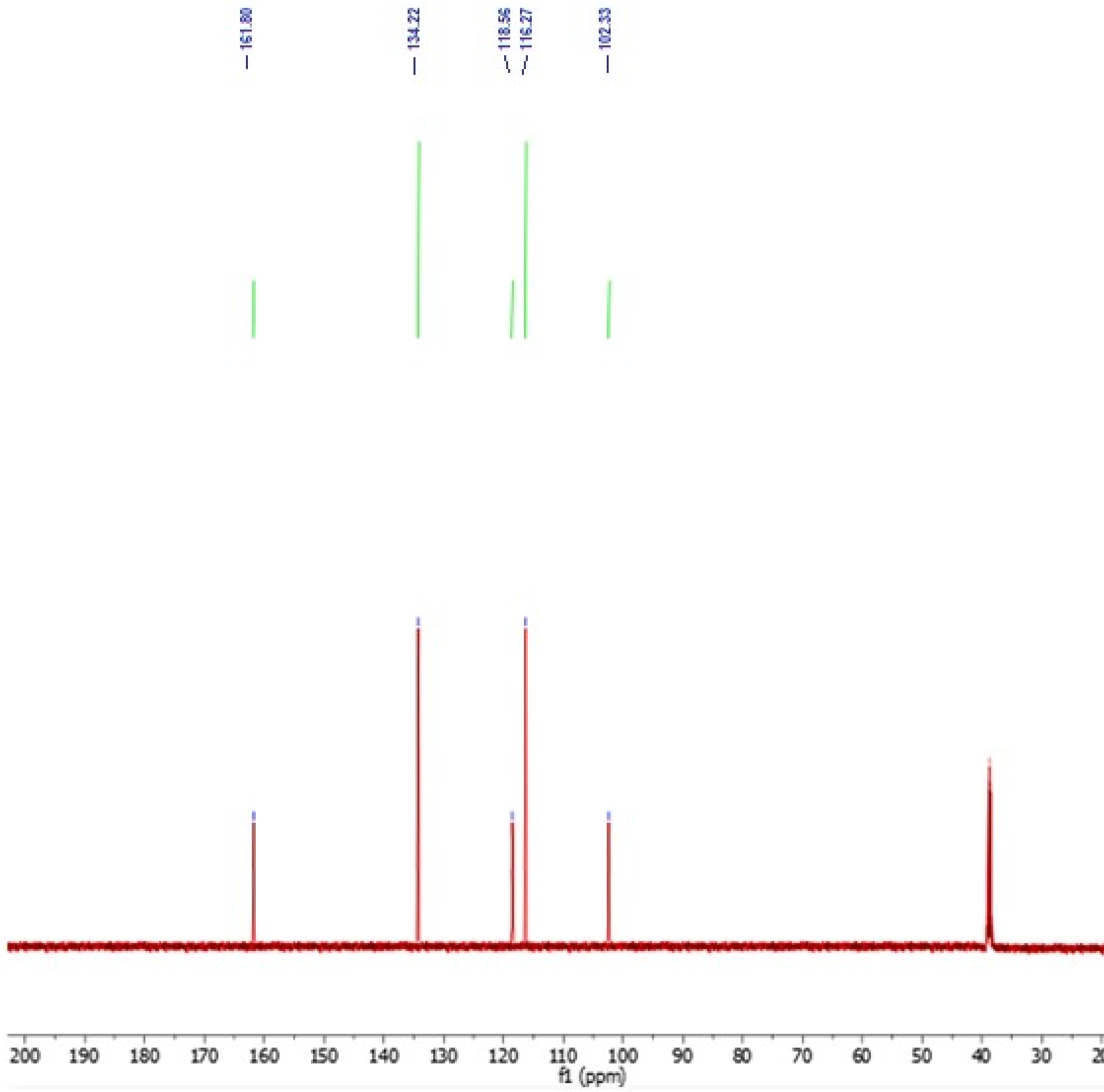

161.80
134.22
118.56
116.27
102.33
200 190 180 170 160 150 140 130 120 110 100 90 80 70 60 50 40 30 20 10 0
f1 (ppm)


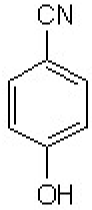

CN
OH

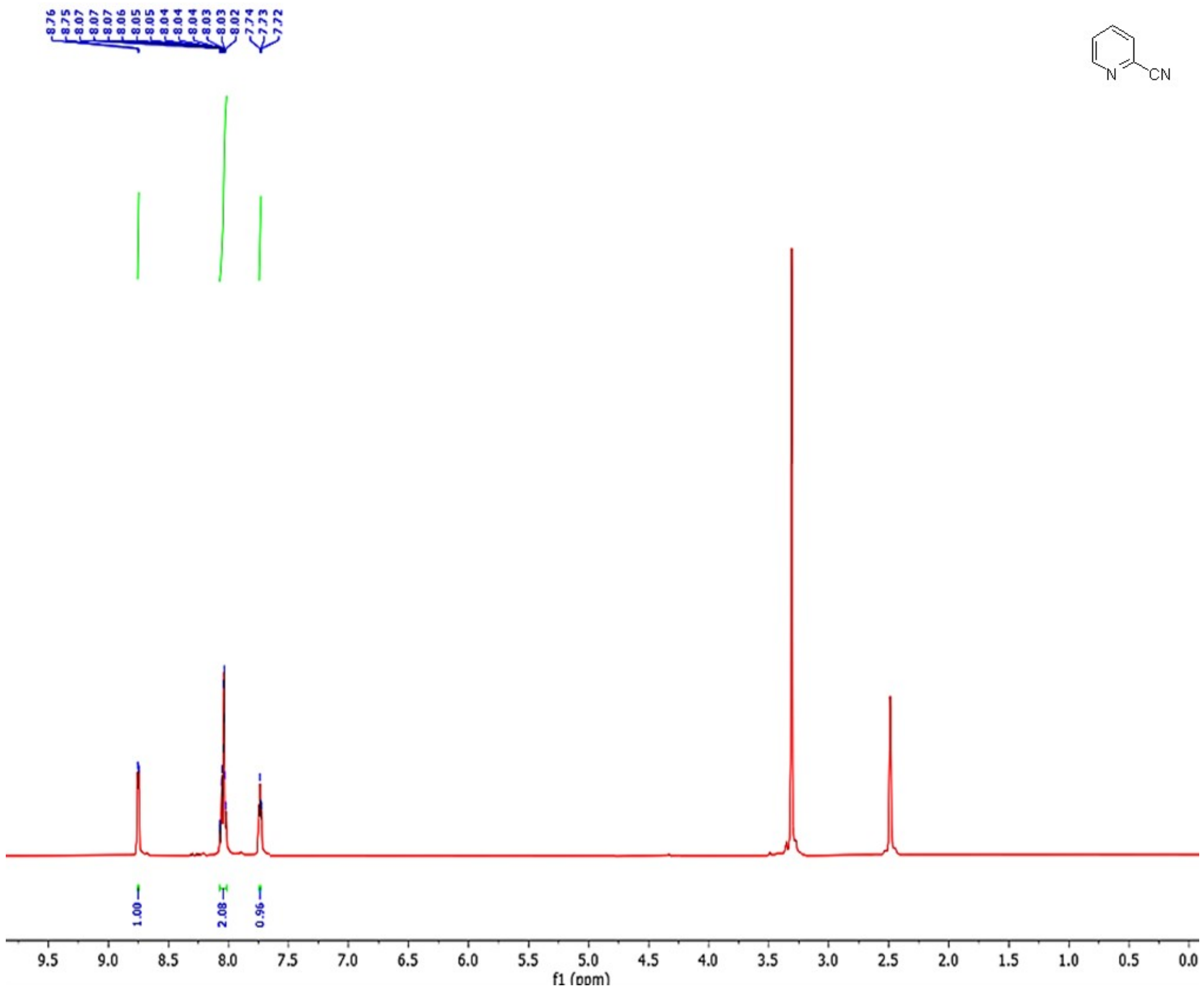

8.76
8.75
8.07
8.07
8.07
8.06
8.05
8.05
8.04
8.04
8.04
8.03
8.03
8.02
7.74
7.73
7.72
N
CN
1.00
2.08
0.96
9.5
9.0
8.5
8.0
7.5
7.0
6.5
6.0
5.5
5.0
4.5
4.0
3.5
3.0
2.5
2.0
1.5
1.0
0.5
0.0
f1 (ppm)

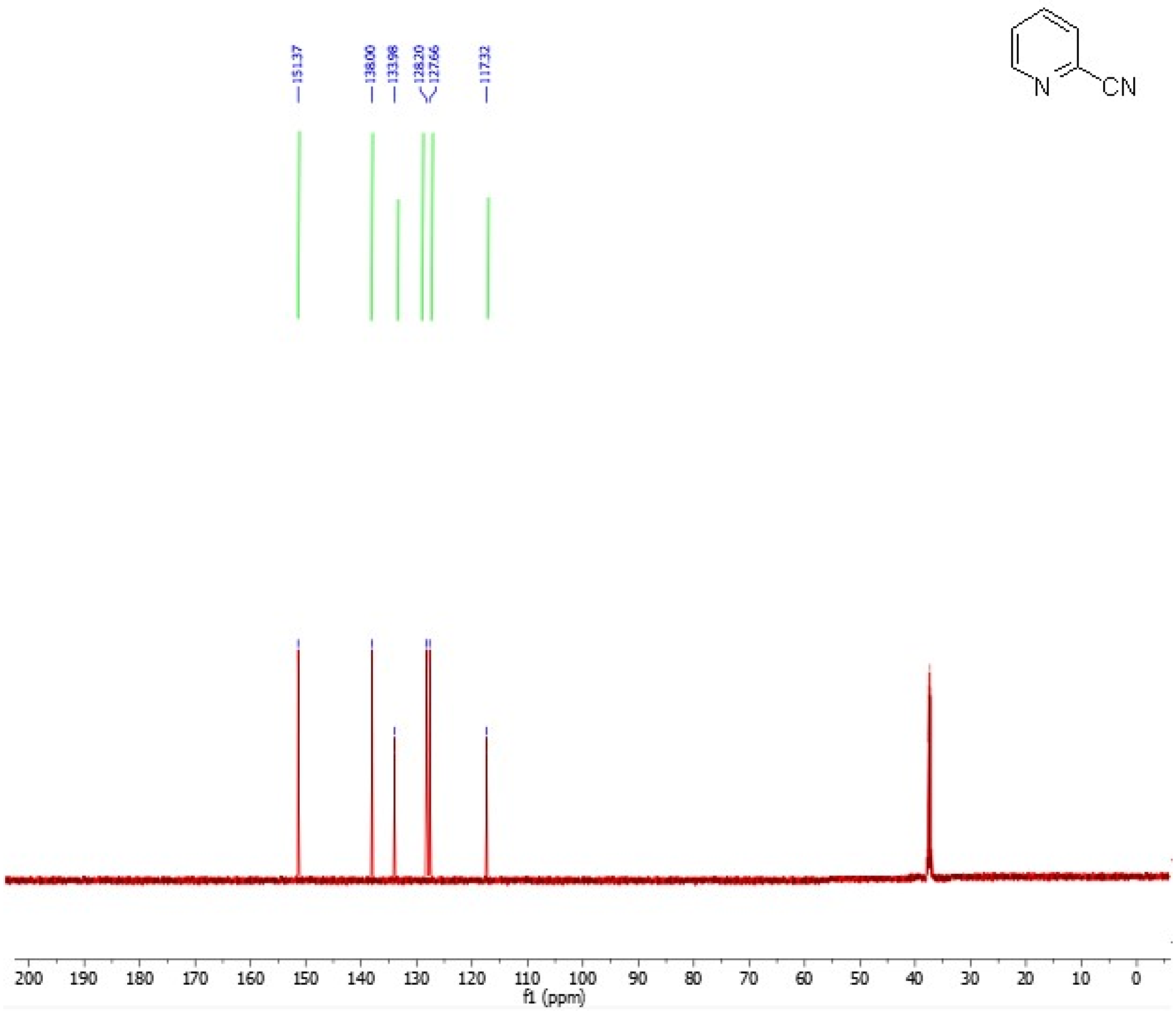

151.37
138.00
133.98
128.20
127.66
117.32
N
CN
200 190 180 170 160 150 140 130 120 110 100 90 80 70 60 50 40 30 20 10 0
f1 (ppm)

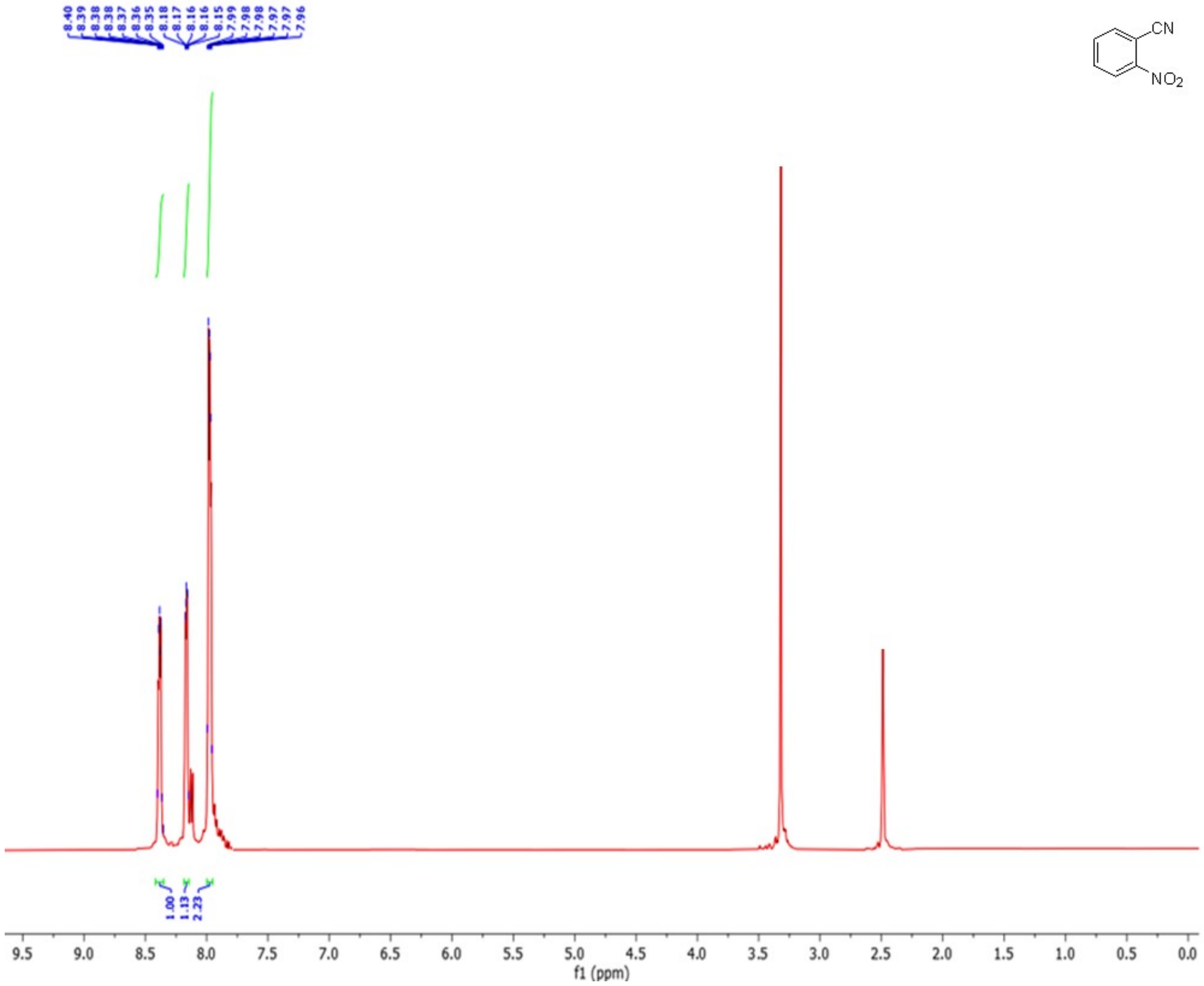
8.40
8.39
8.38
8.38
8.37
8.36
8.35
8.18
8.17
8.16
8.16
8.15
7.99
7.98
7.98
7.97
7.97
7.96
CN
NO2
1.00
1.13
2.23
9.5 9.0 8.5 8.0 7.5 7.0 6.5 6.0 5.5 5.0 4.5 4.0 3.5 3.0 2.5 2.0 1.5 1.0 0.5 0.0
f1 (ppm)

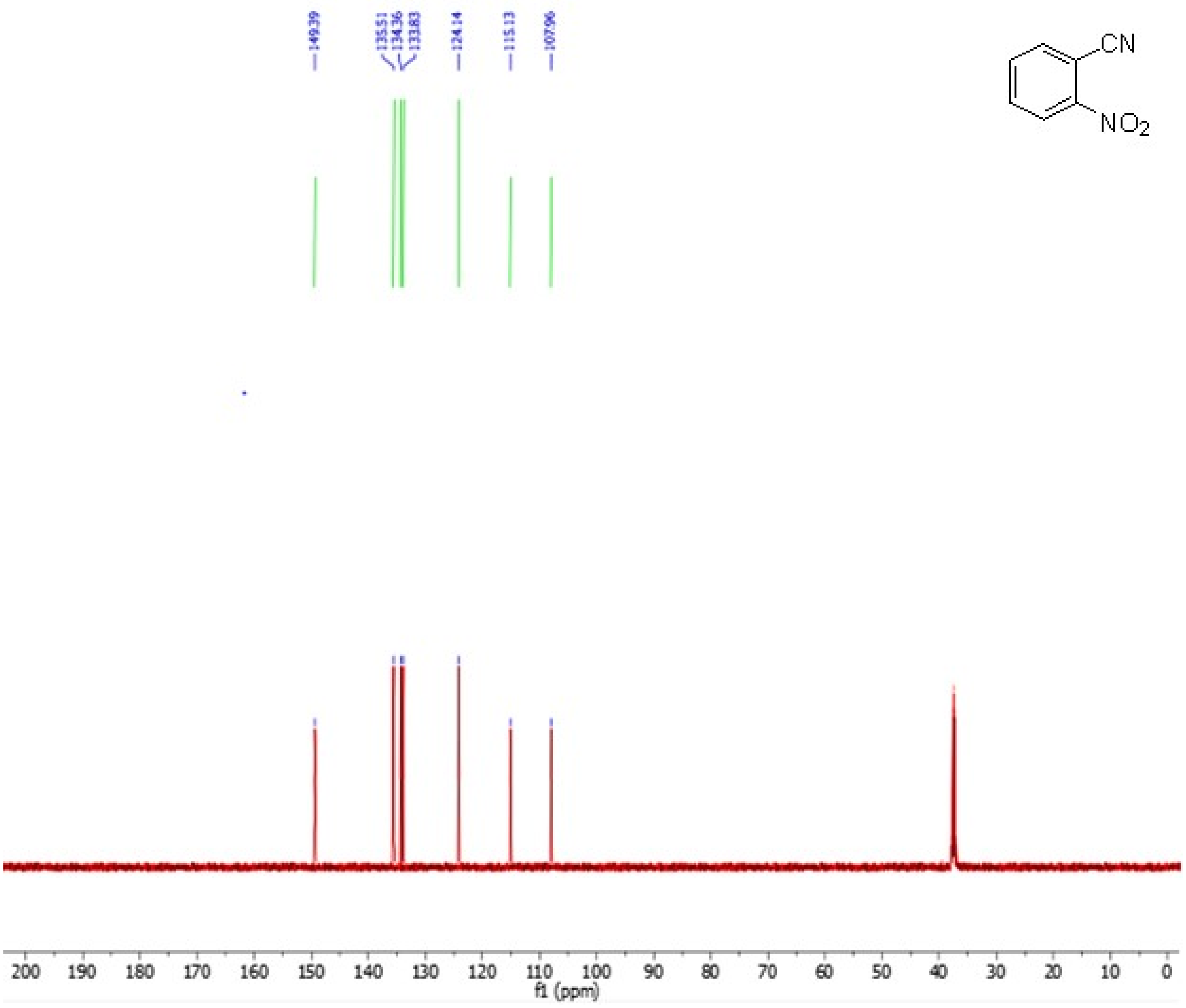
149.39
135.51
134.36
133.83
124.14
115.13
107.96
200 190 180 170 160 150 140 130 120 110 100 90 80 70 60 50 40 30 20 10 0
f1 (ppm)
CN
NO2

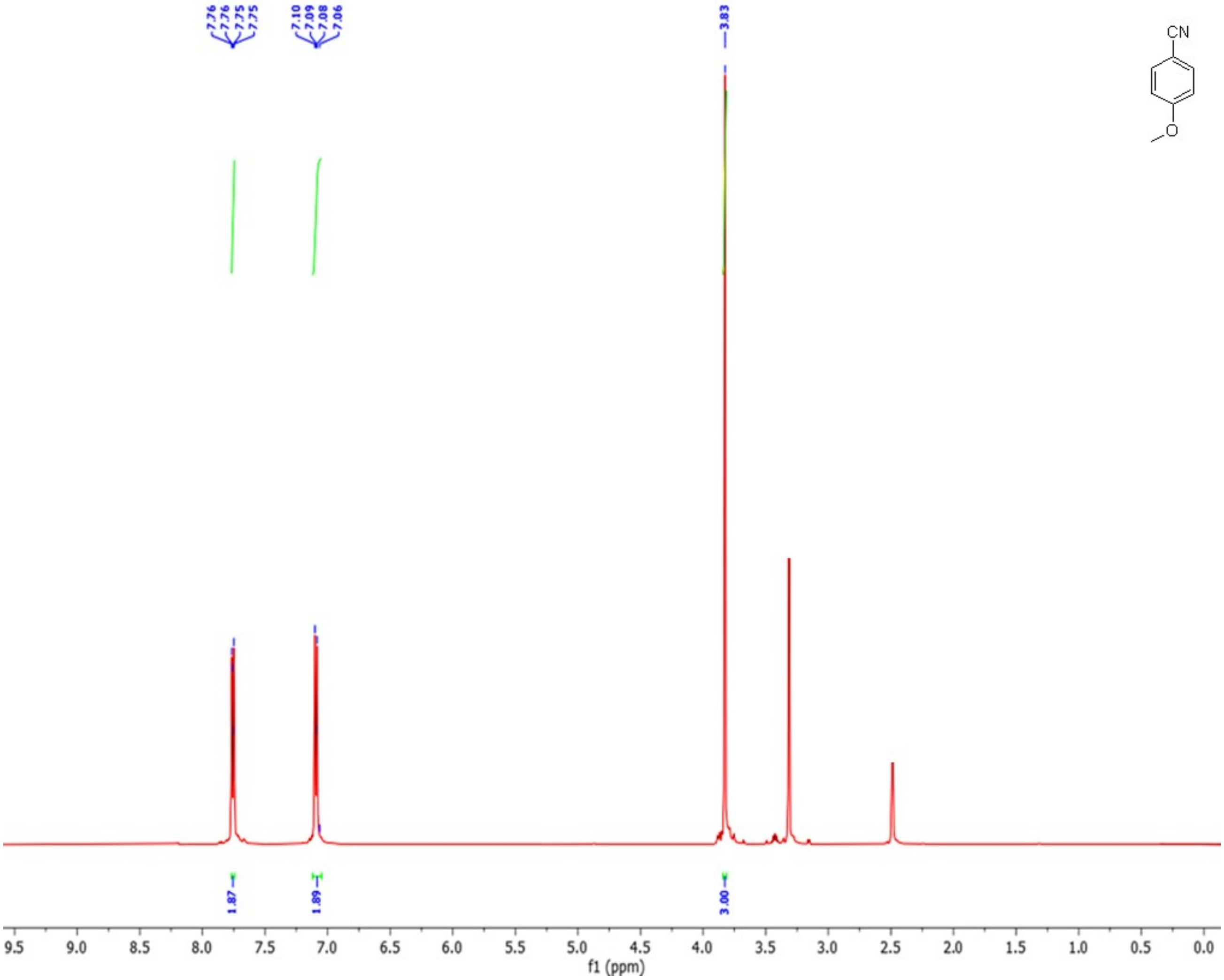
7.76
7.76
7.75
7.75
7.10
7.09
7.08
7.06
3.83
CN
O
1.87
1.89
3.00
9.5
9.0
8.5
8.0
7.5
7.0
6.5
6.0
5.5
5.0
4.5
4.0
3.5
3.0
2.5
2.0
1.5
1.0
0.5
0.0
f1 (ppm)

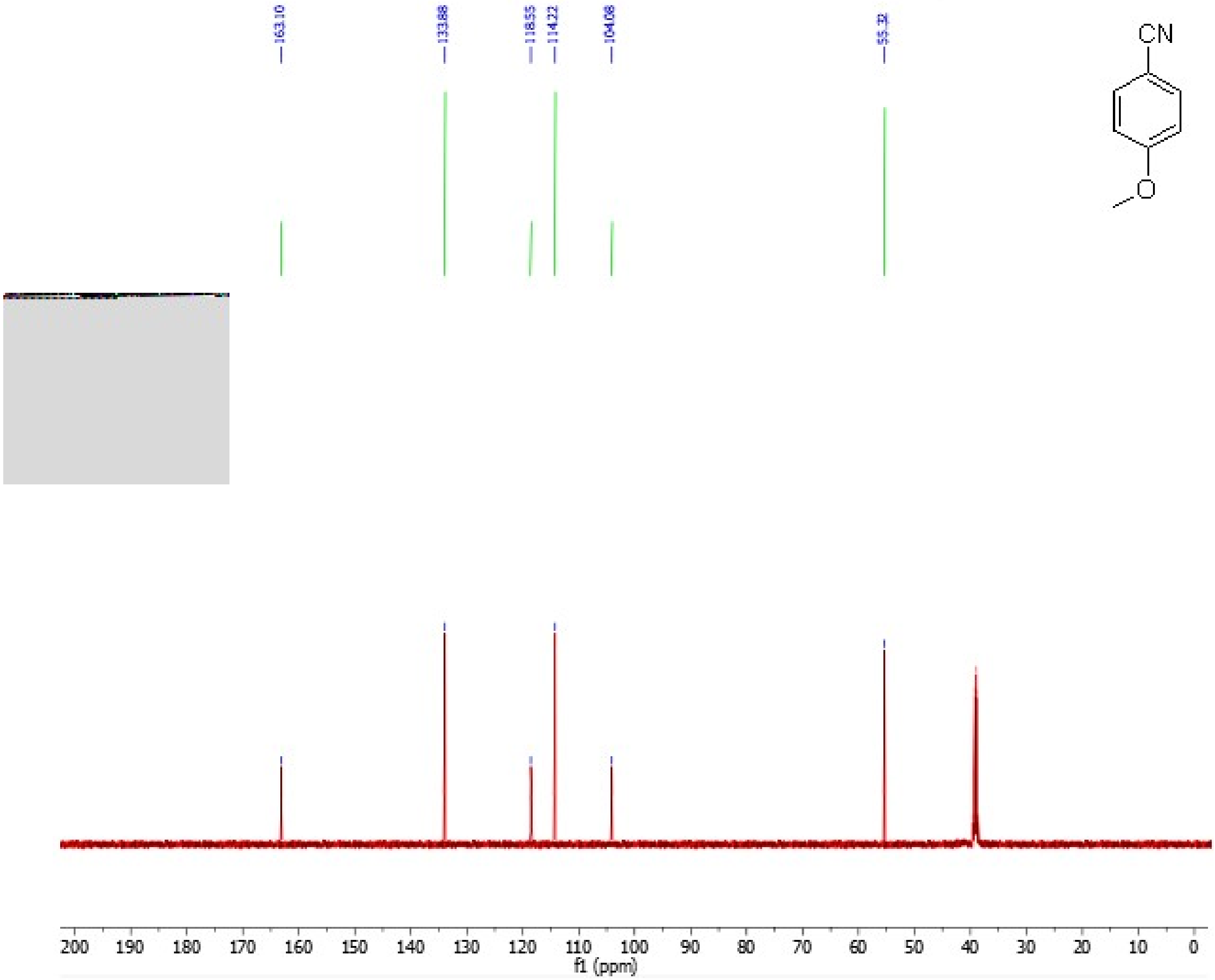
163.10
133.88
118.55
114.22
104.08
55.32
CN
O
200 190 180 170 160 150 140 130 120 110 100 90 80 70 60 50 40 30 20 10 0
f1 (ppm)

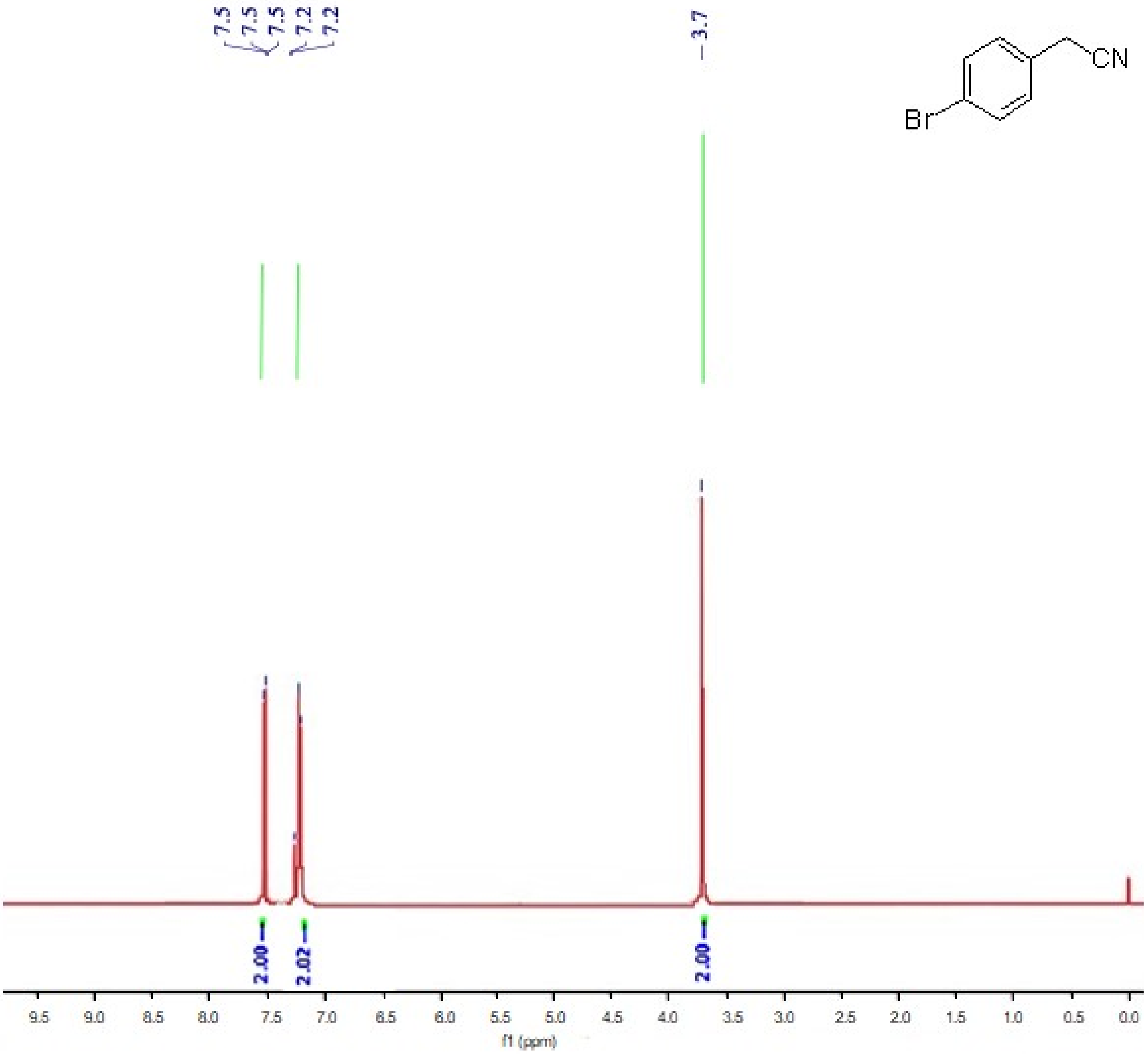
7.5
7.5
7.5
7.2
7.2
3.7
Br
CN
2.00
2.02
2.00
9.5
9.0
8.5
8.0
7.5
7.0
6.5
6.0
5.5
5.0
4.5
4.0
3.5
3.0
2.5
2.0
1.5
1.0
0.5
0.0
f1 (ppm)

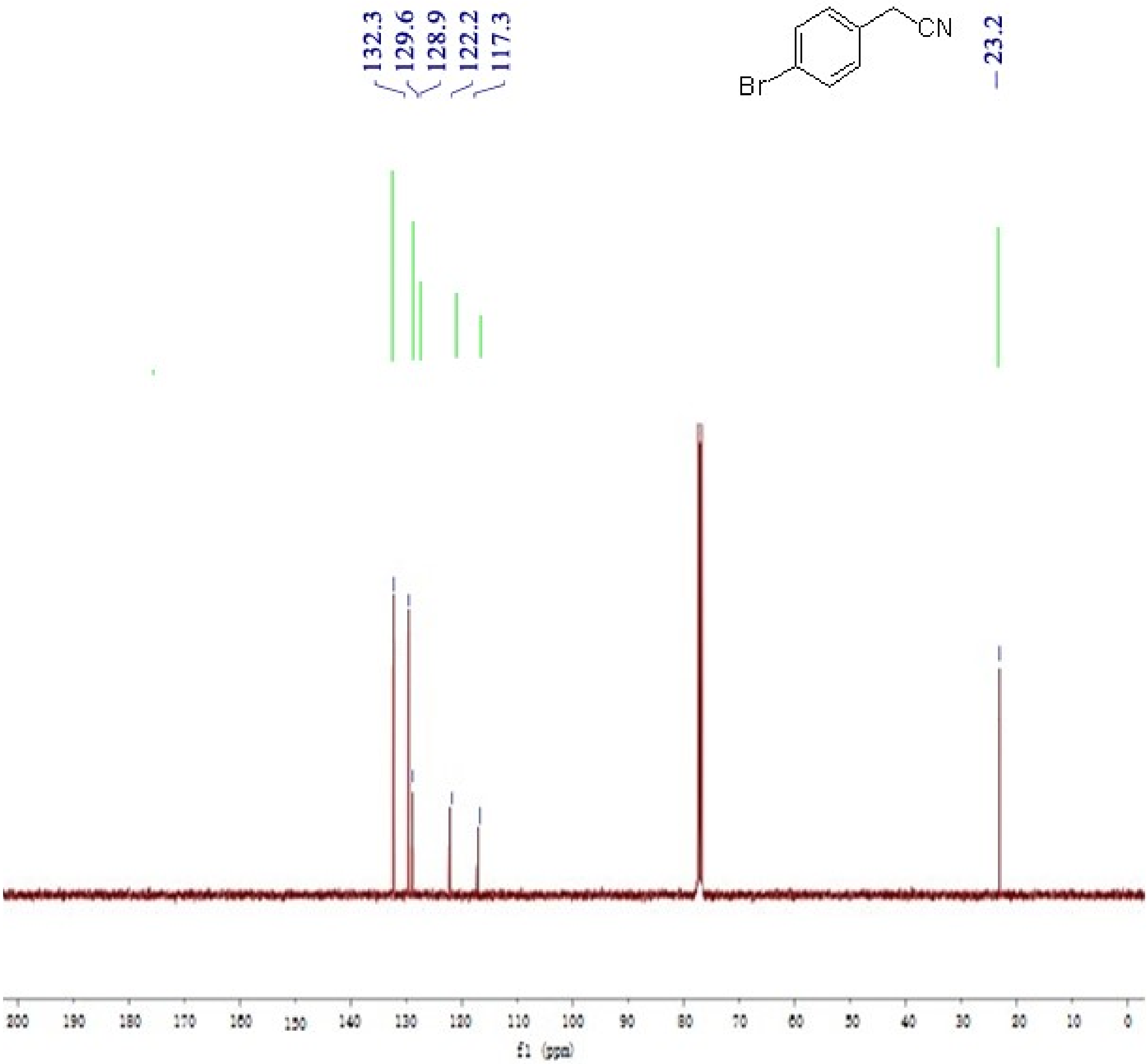
132.3
129.6
128.9
122.2
117.3
Br
CN
23.2
200 190 180 170 160 150 140 130 120 110 100 90 80 70 60 50 40 30 20 10 0
f1 (ppm)

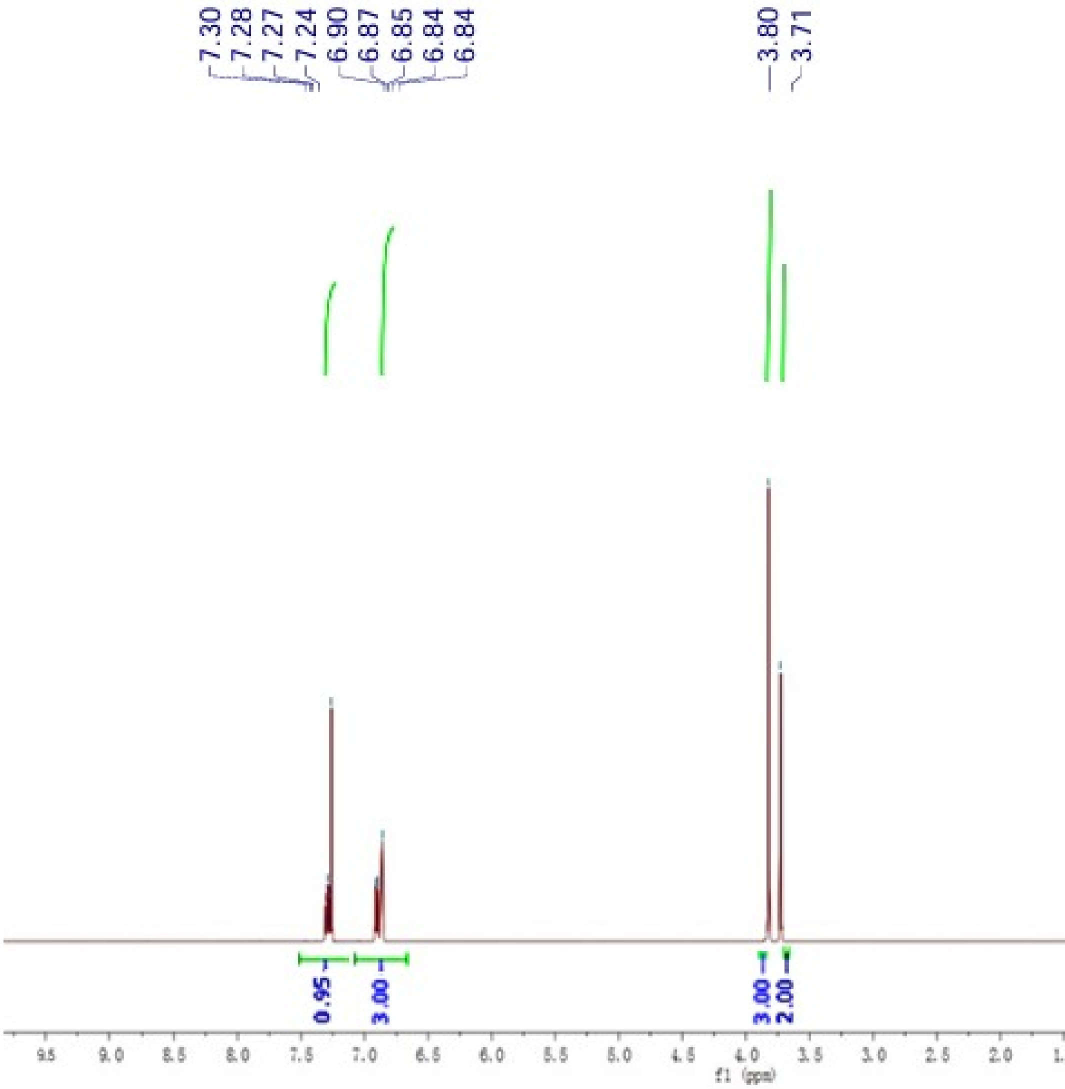

7.30
7.28
7.27
7.24
6.90
6.87
6.85
6.84
6.84
3.80
3.71
0.95
3.00
3.00
2.00
9.5
9.0
8.5
8.0
7.5
7.0
6.5
6.0
5.5
5.0
4.5
4.0
3.5
3.0
2.5
2.0
1.5
1.0
0.5
0.0
f1 (ppm)


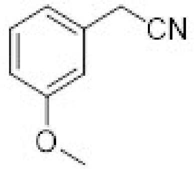

CN
O

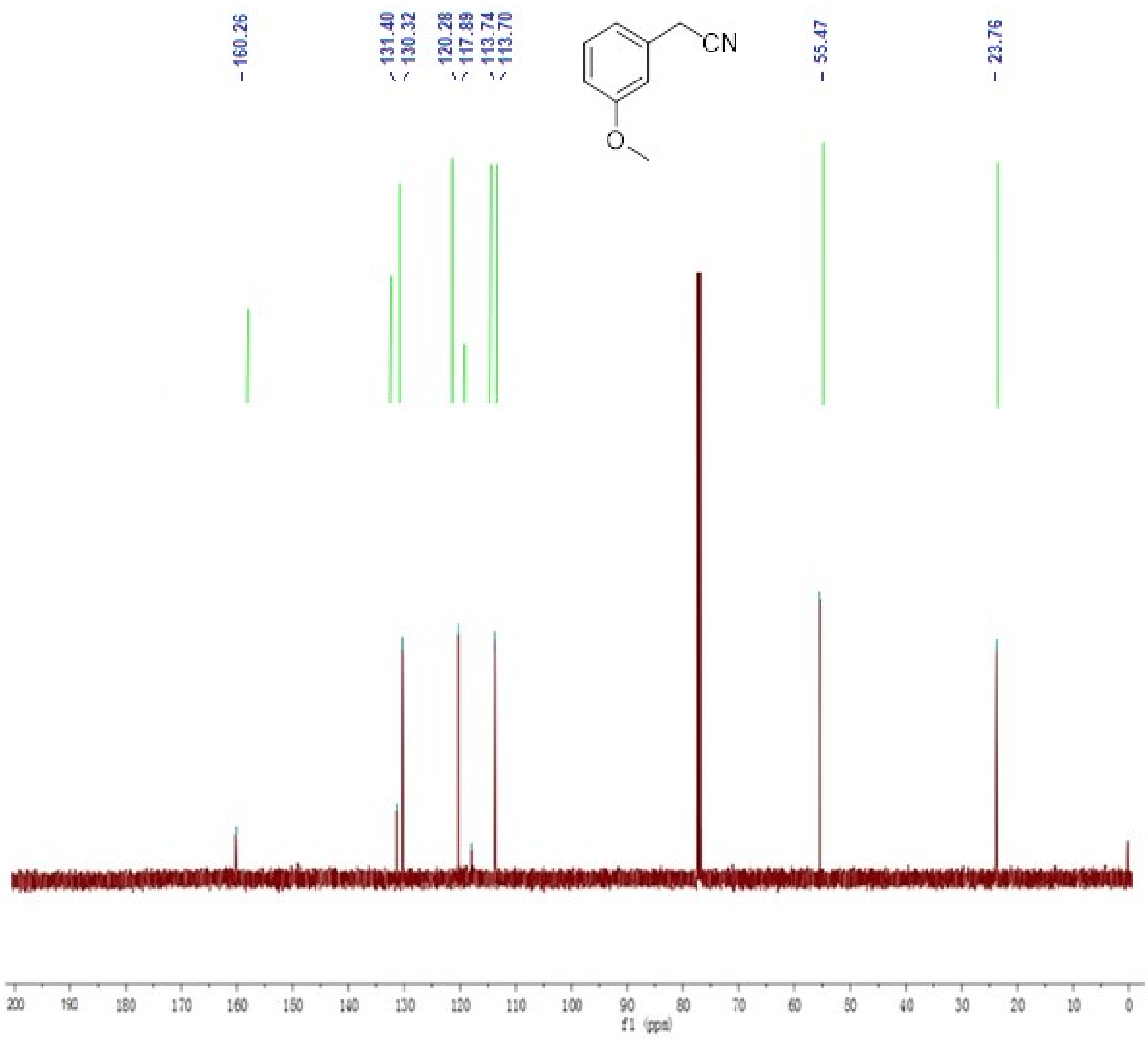
160.26
131.40
130.32
120.28
117.89
113.74
113.70
CN
O
55.47
23.76
200
190
180
170
160
150
140
130
120
110
100
90
80
70
60
50
40
30
20
10
0
f1 (ppm)

**References:**

Shimojo, Hiroyuki; Moriyama, Katsuhiko; Togo, Hideo, Synthesis, 2013, 45, 2164.

Dighe, Shashikant U.; Chowdhury, Deepan; Batra, Sanjay, Advanced Synthesis and Catalysis, 2014, 356, 3892 – 3896.

Han, Min Su; Jang, Mingyeong; Lim, Taeho; Park, Byoung Yong, Journal of Organic Chemistry, 2022, 87, 910 – 919.

Xu, Da Peng; Xiong, Meilu; Kazem nejadi, Milad, RSC Advances, 2021, 11, 21, 12484 – 12499.
